# Dysregulated mitochondrial metabolism upon cigarette smoke exposure in various human bronchial epithelial cell models

**DOI:** 10.1242/dmm.049247

**Published:** 2022-03-28

**Authors:** Christy B. M. Tulen, Ying Wang, Daan Beentjes, Phyllis J. J. Jessen, Dennis K. Ninaber, Niki L. Reynaert, Frederik-Jan van Schooten, Antoon Opperhuizen, Pieter S. Hiemstra, Alexander H. V. Remels

**Affiliations:** 1 Department of Pharmacology and Toxicology, School of Nutrition and Translational Research in Metabolism, Maastricht University Medical Center+, PO Box 616, 6200 MD Maastricht, The Netherlands; 2 Department of Pulmonology, Leiden University Medical Center, PO Box 9600, 2300 RC Leiden, The Netherlands; 3 Department of Respiratory Medicine, School of Nutrition and Translational Research in Metabolism, Maastricht University Medical Center+, PO Box 616, 6200 MD Maastricht, The Netherlands; 4 Primary Lung Culture Facility, Maastricht University Medical Center+, PO Box 616, 6200 MD Maastricht, The Netherlands; 5 Office of Risk Assessment and Research, Netherlands Food and Consumer Product Safety Authority, PO Box 8433, 3503 RK Utrecht, The Netherlands

**Keywords:** Autophagy, Cell model, Cigarette smoke, Culture methods, Human primary bronchial epithelial cells, Mitochondrial metabolism

## Abstract

Exposure to cigarette smoke (CS) is the primary risk factor for developing chronic obstructive pulmonary disease. The impact of CS exposure on the molecular mechanisms involved in mitochondrial quality control in airway epithelial cells is incompletely understood. Undifferentiated or differentiated primary bronchial epithelial cells were acutely/chronically exposed to whole CS (WCS) or CS extract (CSE) in submerged or air–liquid interface conditions. Abundance of key regulators controlling mitochondrial biogenesis, mitophagy and mitochondrial dynamics was assessed. Acute exposure to WCS or CSE increased the abundance of components of autophagy and receptor-mediated mitophagy in all models. Although mitochondrial content and dynamics appeared to be unaltered in response to CS, changes in both the molecular control of mitochondrial biogenesis and a shift toward an increased glycolytic metabolism were observed in particular in differentiated cultures. These alterations persisted, at least in part, after chronic exposure to WCS during differentiation and upon subsequent discontinuation of WCS exposure. In conclusion, smoke exposure alters the regulation of mitochondrial metabolism in airway epithelial cells, but observed alterations may differ between various culture models used.

This article has an associated First Person interview with the joint first authors of the paper.

## Abstract (precis)

**Summary:** Exposure of primary human bronchial epithelial cells to smoke affected mitochondria differently in various exposure and culture models, highlighting the importance of tailoring the model to the research question.

## INTRODUCTION

Exposure to cigarette smoke (CS) is the most important risk factor for developing chronic obstructive pulmonary disease (COPD), a leading cause of mortality and burden of disease worldwide (Celli and Wedzicha, 2019; WHO, 2020). However, whereas much has been learnt about the role of oxidative stress and CS-induced inflammation, our insight into the molecular mechanisms driving smoke-induced COPD pathogenesis still has various knowledge gaps, including those related to mitochondrial function.

Recent studies have suggested a crucial role for mitochondrial dysfunction in the pathogenesis of smoking-related lung diseases such as COPD (Aghapour et al., 2020; Cloonan and Choi, 2016; Cloonan et al., 2016; Hiemstra and van der Does, 2017; Pan et al., 2019; Prakash et al., 2017). Indeed, a variety of studies have identified abnormal mitochondrial morphology, e.g. swelling and fragmentation, in airway epithelial cells of COPD patients, which was recapitulated in various *in vivo* and *in vitro* experimental exposure models with CS or CS extract (CSE), respectively (Agarwal et al., 2014; Cloonan et al., 2016; Hara et al., 2013; Hoffmann et al., 2013; Malinska et al., 2018; Mizumura et al., 2014; Sundar et al., 2019; Valdivieso et al., 2018; van der Toorn et al., 2009; Wu et al., 2020). Moreover, in mice, amelioration of CS-induced mitochondrial dysfunction alleviated COPD-associated pathological features (Cloonan et al., 2016).

Mitochondrial function and content are regulated by a series of crucial cellular quality control processes, including mitochondrial biogenesis and mitophagy. Mitochondrial biogenesis is essentially regulated via the peroxisome proliferator-activated receptor gamma, coactivator 1 (PPARGC1) signaling network, which includes a myriad of transcription factors and transcriptional co-activators (Jornayvaz and Shulman, 2010; Lin et al., 2005; Scarpulla, 2011) that cooperatively drive the genesis of new organelles. On the other hand, defective/damaged mitochondria can be degraded by selective autophagy, i.e. mitophagy, which is modulated by two specific pathways (Gomes and Scorrano, 2013): (1) receptor-mediated mitophagy, initiated by mitochondrial receptors, such as BCL2/adenovirus E1B 19 kDa protein-interacting protein 3 (BNIP3), BNIP3-like (BNIP3L) and FUN14 domain-containing 1 (FUNDC1); and (2) ubiquitin-mediated mitophagy, triggered by loss of mitochondrial membrane potential, accumulation of PTEN-induced kinase 1 (PINK1) and recruitment of parkin RBR E3 ubiquitin protein ligase (PRKN) in the outer mitochondrial membrane. Evidently, both mitophagy pathways require general autophagy proteins, such as GABA type A receptor-associated protein-like 1 (GABARAPL1), microtubule-associated protein 1B light chain 3 (MAP1LC3) alpha and beta, and sequestosome 1 (SQSTM1), to facilitate formation of the autophagosomal membrane around the mitochondrion (Fritsch et al., 2020).

Autophagy has been suggested to be critically involved in COPD pathogenesis (Hikichi et al., 2019). Previous research indicated that markers of autophagy and mitophagy are induced in lung tissue of COPD patients (Ahmad et al., 2015; Chen et al., 2008; Ito et al., 2015; Mizumura et al., 2014). This was confirmed in various models of CS(E) exposure of (human) airway epithelial cells; however, most studies deployed cell lines or submerged undifferentiated cultures of primary human airway epithelial cells (Ahmad et al., 2015; Chen et al., 2008; Hoffmann et al., 2013; Ito et al., 2015; Kyung et al., 2018; Mizumura et al., 2014, 2018; Park et al., 2019; Son et al., 2018; Song et al., 2017; Wu et al., 2020; Zhang et al., 2019a), which are not an accurate representation of the complex *in vivo* cellular architecture of the human airway epithelium.

Because epithelial cells that line the respiratory tract are the first cells to be exposed to inhaled toxicants, the relevance of studying the impact of exposure to CS on mitochondrial function and content in airway epithelial cells is obvious (Hiemstra et al., 2018). However, the epithelial lining of the airways consists of several distinct cell types, not all of which are included in most studied cell lines or submerged culture models. Studying the variety of cell types is essential because individual cell types differ in number and intracellular organization of mitochondria, which may be related to their function and corresponding energy demand (Aghapour et al., 2020). Therefore, a differentiated pseudostratified layer of primary airway epithelial cells, including ciliated, club and goblet cells, is needed to study these processes in culture models (Mertens et al., 2017). However, previous studies used cell lines or submerged undifferentiated primary bronchial epithelial cells (PBEC) (more representative of a basal-like cell type reflecting damaged epithelium) to assess the impact of CSE on autophagy and mitophagy (Ahmad et al., 2015; Chen et al., 2008; Hoffmann et al., 2013; Ito et al., 2015; Kyung et al., 2018; Mizumura et al., 2014, 2018; Park et al., 2019; Son et al., 2018; Song et al., 2017; Wu et al., 2020; Zhang et al., 2019a). Applying a more physiologically relevant model consisting of the various epithelial cell types differentiated by culture at the air–liquid interface (ALI), which allows exposure to whole CS (WCS) (including gaseous and particulate mainstream CS components) instead of an aqueous CSE, has the potential to provide better insight into the impact of CS on the regulation of critical mitochondrial quality control processes. Importantly, we previously reported that differentiation of PBEC at the ALI is accompanied by a marked increase in expression of genes involved in xenobiotic metabolism and increases metabolic activity (Boei et al., 2017). This is relevant for studying effects of CS exposure on PBEC cultures, because such biotransformation reactions may contribute to both detoxification of CS components as well as to formation of (more) reactive species. Therefore, comparison of such more advanced models to more simple conventional models using submerged cultures and CSE is needed to establish whether or not the use of more complex models is warranted over the use of more simple *in vitro* models.

In addition to these considerations, most available *in vitro* studies evaluated the impact of acute CS(E) exposure on mitochondrial quality control systems in airway epithelial cells. Given that COPD develops as a result of chronic exposure to CS, it is important to characterize the impact of acute versus chronic CS exposure on these cellular processes. Moreover, only a few *in vitro* studies investigated the long-lasting impact on parameters relevant to COPD pathophysiology after termination of CS exposure. These studies demonstrated recovery of epithelial differentiation markers (Amatngalim et al., 2018), while inflammation (Rutgers et al., 2000; Wang et al., 2018b), apoptosis (Hodge et al., 2005) and alterations in mitochondrial morphology and function (Hoffmann et al., 2013) have been shown to persist upon smoking cessation *in vivo* in epithelial cells derived from former-smoking COPD patients as well as in *in vitro* airway culture models. However, the impact of smoking cessation on mechanisms that underlie these (CS-induced) mitochondrial aberrations (i.e. mitochondrial biogenesis and mitophagy) are incompletely understood. In addition, whether, and to what extent, changes in these processes persist after smoking cessation is unknown.

Therefore, our study investigated, for the first time, the abundance of a comprehensive panel of key regulatory molecules involved in mitochondrial metabolism in various *in vitro* models of CS exposure using human PBEC from multiple (non-COPD) donors (*n*=3/4 donors/model). These cells were either cultured and exposed in a submerged, undifferentiated status or were differentiated by culture in an ALI culture system and subsequently exposed to CSE or WCS, respectively. In addition to assessing the acute response of mitochondrial quality control processes to CS exposure, we also studied the impact of repeated/chronic WCS exposure during differentiation as well as recovery of mitochondria following cessation of WCS exposure in our ALI-PBEC (undifferentiated PBEC cultured at the ALI to induce differentiation) model. This way, we obtained insight into differences and similarities between the effects of CS on the regulation of mitochondrial metabolism in four different epithelial culture models.

## RESULTS

**Fig. 1.**
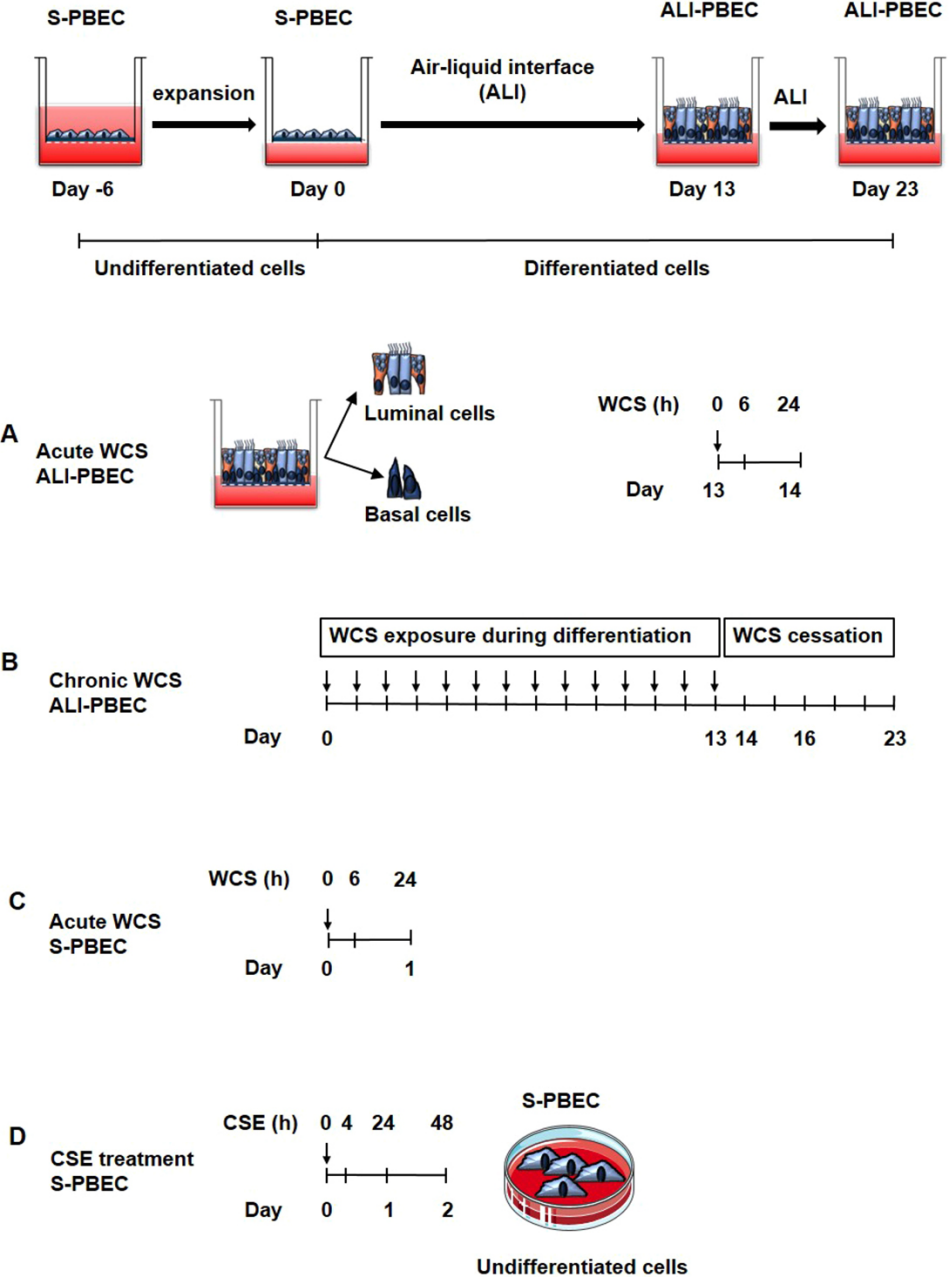
**Experimental cigarette smoke (CS)-exposed cell models.** Models A, B and C were primary bronchial epithelial cells (PBEC) cultured on transwells (see timeline of expansion and differentiation at the top of the figure) to allow apical exposure to fresh air or whole CS (WCS), whereas the model shown in D was PBEC cultured on tissue culture plastic requiring submerged exposure to CS extract (CSE). (A) Acute WCS-exposed ALI-PBEC: after 2 weeks of differentiation, ALI-PBEC [undifferentiated PBEC cultured at the air–liquid interface (ALI) to induce differentiation] were exposed to fresh air or WCS from one 3R4F cigarette (University of Kentucky, 2 mg). Subsequently, whole-cell lysates were harvested after 6 h and 24 h, and the basal and luminal fractions were harvested only at 6 h post-exposure (*n*=3 donors/group). (B) Chronic WCS-exposed (during differentiation) ALI-PBEC followed by smoking cessation: ALI-PBEC were 1× daily exposed to fresh air or WCS from one 3R4F cigarette (University of Kentucky, 2 mg) during differentiation for 14 days followed by a cessation period up to 10 days. Cells were harvested on Day 14 (24 h after the last WCS exposure), 16, 19 and 23 (*n*=3 donors/group). (C) Acute WCS-exposed S-PBEC: undifferentiated S-PBEC (PBEC cultured in submerged conditions) were exposed, after removal of apical medium, to fresh air or WCS from one 3R4F cigarette (University of Kentucky, 2 mg), followed by harvesting of whole-cell lysates after 6 h and 24 h recovery (*n*=3 donors/group). (D) CSE-treated S-PBEC: undifferentiated S-PBEC were submerged treated with CSE from one 3R4F cigarette (University of Kentucky) diluted in HBSS (0-1-2%) in Lonza starvation medium for 4, 24 or 48 h (*n*=4 donors/group).

We deployed four models of exposure of PBEC to WCS or CSE. These included, respectively, (1) differentiated ALI-PBEC acutely exposed to WCS, (2) ALI-PBEC chronically exposed (during differentiation) to WCS followed by smoking cessation, (3) undifferentiated S-PBEC (PBEC cultured in submerged conditions) acutely exposed to WCS and (4) undifferentiated S-PBEC treated with CSE (Fig. 1). ALI-PBEC cultures were differentiated and included several distinct cell types present in the pseudostratified epithelium, mimicking the ‘healthy’ normal airway epithelium (Amatngalim et al., 2018). Undifferentiated S-PBEC, on the other hand, consisted of basal cells, reflecting injured/damaged airway epithelium. Collectively, these models allowed us not only to test our hypothesis that WCS exposure has a differential impact on mitochondrial quality control systems in undifferentiated (predominantly basal-like cell type) versus differentiated (including ciliated, club and goblet cells) human PBEC cultures, but also allowed us to compare the effect of CSE to that of WCS (particles and gaseous components) and assess the potential influence of smoking cessation.

### Increase in autophagy markers in response to CS exposure

As autophagy proteins play a key role in facilitating mitochondrial quality control (i.e. breakdown) by the autophagosomal pathways, we studied the effect of WCS exposure in ALI-PBEC on the expression of several autophagy-related proteins. First, as it has been reported that activation of autophagy, as a cytoprotective mechanism, is related to oxidative stress (Yun et al., 2020), and to verify that our CS exposures were able to elicit a cellular response indicative of successful exposure in the different models, we investigated the oxidative stress response to WCS or CSE by measuring antioxidant gene expression in our four models. Elevated mRNA levels of superoxide dismutase 1 (*SOD1*) were observed in ALI-PBEC following acute WCS exposure and 24 h after chronic WCS exposure during differentiation (Fig. S1A,C). Separation of cellular fractions revealed that the acute WCS-induced upregulation of *SOD2* expression originated from both the luminal and basal cell fraction (Fig. S1B). The separation of basal and luminal cell fractions was validated by measuring gene expression of the basal cell marker *TP63* and early progenitor cell marker *KRT8* (Fig. S2), respectively. In contrast to changes observed in ALI-PBEC exposed to WCS, in S-PBEC exposed to CSE only, *SOD1* transcript levels were induced (in a dose-dependent manner) whereas *SOD2* transcript levels were decreased, which was not observed in S-PBEC exposed to WCS (Fig. S1D).

Our previous results also showed that acute WCS exposure resulted in increased expression of genes involved in the antioxidant defense, including heme oxygenase 1 and NAD(P)H quinone oxidoreductase 1 in ALI-PBEC (Zarcone et al., 2016). In accordance with these previous data, our results collectively point to a cellular response to oxidative stress indicative of successful exposure to CS(E) in the different models.

**Fig. 2.**
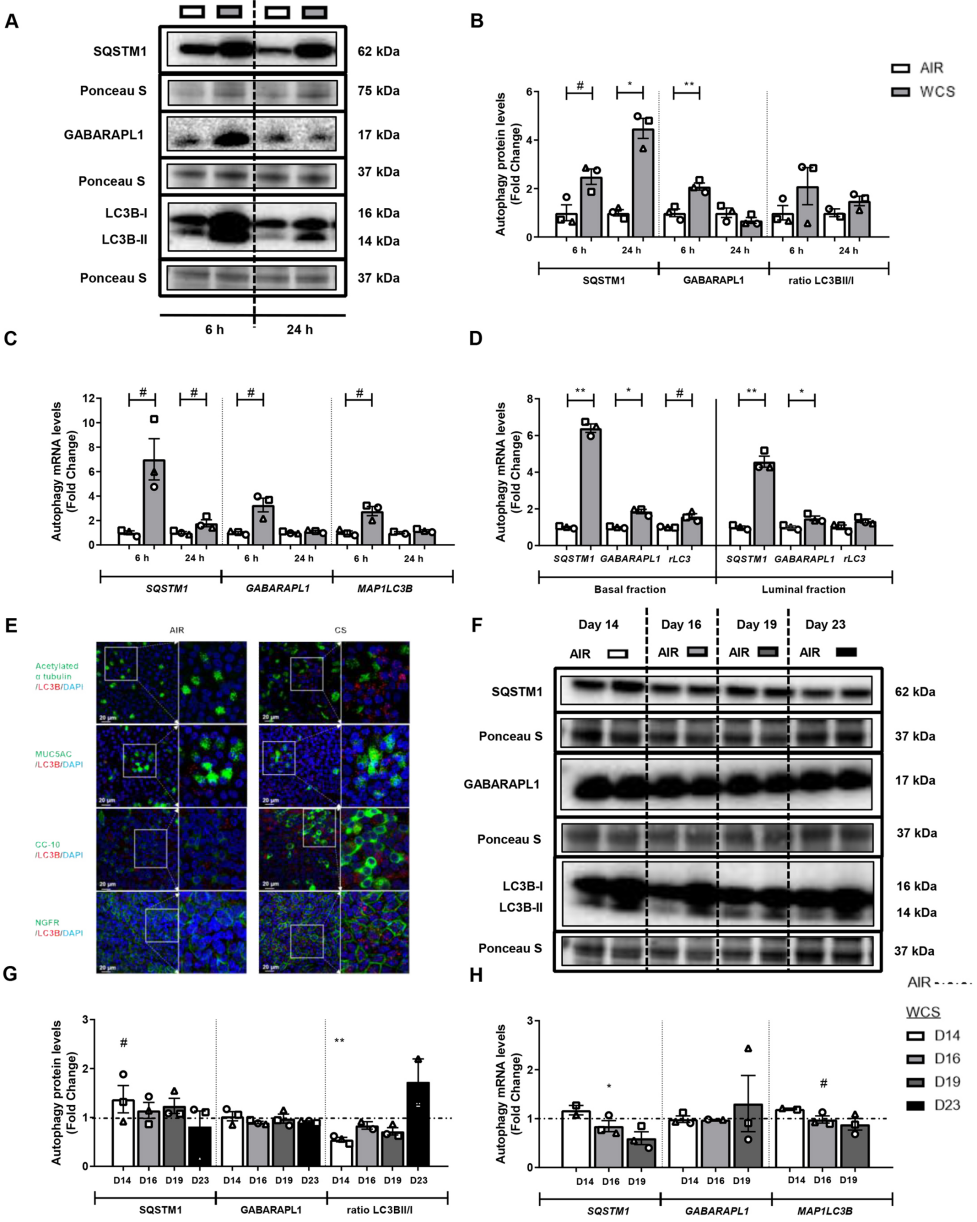
**Increase in abundance of autophagy markers in WCS-exposed ALI-PBEC.** After 2 weeks of differentiation, ALI-PBEC were exposed to fresh air or WCS from one 3R4F cigarette (University of Kentucky, 2 mg), and whole-cell lysates were harvested after 6 h and 24 h, and the basal and luminal fractions were harvested only at 6 h post-exposure (*n*=2-3 donors/group). (A-D) Protein (A,B) and transcript (C,D) levels of autophagy regulators SQSTM1, GABARAPL1, MAP1LC3B and ratios of LC3BII/I or MAP1LC3B/A (rLC3) in whole-cell lysates or in basal/luminal cell fractions post-exposure are presented. Data are presented as mean fold change compared to control (air)±s.e.m. Independent donors are represented by open circles, triangles or squares. Statistical differences between WCS versus air were tested using a two-tailed paired parametric *t*-test (^#^*P*<0.1, *P<0.05 and **P<0.01). (E) Immunofluorescence staining and confocal microscopy were conducted post-WCS exposure in differentiated ALI-PBEC using antibodies for anti-LC3B (red) together with anti-MUC5AC (green), anti-acetylated α-tubulin (green), anti-CC-10 (green) and anti-NGFR (green) in combination with 4′,6-diamidino-2-phenylindole (DAPI; blue) for nuclear staining. Immunofluorescence images shown are representative for three donors with 630× original magnification. Scale bars: 20 μm. ALI-PBEC were 1× daily exposed to fresh air or WCS from one 3R4F cigarette (University of Kentucky, 2 mg) during differentiation for 14 days followed by a cessation period of up to 10 days. Cells were harvested on Day 14 (24 h after the last exposure), 16, 19 and 23 (*n*=2-3 donors/group). (F-H) Protein (F,G) and mRNA levels (H) of autophagy regulators SQSTM1, GABARAPL1 and ratio LC3BII/I or MAP1LC3B were analyzed in whole-cell lysates. Representative western blots, including representative parts of the Ponceau S staining, are shown. Data are presented as mean fold change compared to control (air or WCS Day 14)±s.e.m. Independent donors are represented by open circles, triangles or squares. Statistical differences between WCS and air after smoking cessation in ALI-PBEC on each day were tested using a two-tailed paired parametric *t*-test (e.g. WCS Day 14 versus air). Comparison of various groups to test the difference between WCS Day 16, 19, 23 and Day 14 in WCS chronic smoking cessation experiments was conducted using one-way ANOVA followed by Sidak's post-hoc test for multiple comparisons, and, in the case of missing values, mixed-effects modeling was performed. Statistical significance is indicated as ^#^*P*<0.1, *P<0.05 and **P<0.01 compared to control (air or WCS Day 14).

Next, we assessed the impact of exposure of the cells to CS constituents on the expression of autophagy-related proteins. As shown in Fig. 2A-C, both protein and transcript levels of the adaptor proteins SQSTM1 and GABARAPL1, as well as the marker of the accumulation of autophagosomes (LC3BII; also known as MAP1LC3B2), were increased after WCS exposure in ALI-PBEC. These increases were most pronounced at 6 h post-WCS exposure and were largely dissipated after 24 h. Similar results were obtained in S-PBEC exposed to WCS or CSE (Fig. S3), although it appeared that induction of transcript expression of autophagy markers in response to CSE was delayed and more pronounced compared to the changes in ALI-PBEC cultures stimulated with WCS (Fig. S3C). Importantly, to further determine the cellular location of the increased expression of these autophagy markers in response to WCS, we isolated basal and luminal cells from WCS-exposed ALI-PBEC cultures. By comparison, both basal and luminal cell fractions of ALI-PBEC expressed higher mRNA levels of autophagy markers after WCS exposure, indicating that both fractions were similarly affected by WCS exposure (although responses appeared to be slightly more pronounced in the basal fraction) (Fig. 2D). To further investigate and validate these findings, an immunofluorescence assay was conducted, using staining with the anti-LC3B (also known as MAP1LC3B) antibody as autophagy indicator; co-staining with anti-MUC5AC, anti-acetylated α-tubulin, anti-CC-10 (also known as SCGB1A1) and anti-NGFR was used to detect goblet cells, ciliated cells, club cells and basal cells, respectively. As depicted in Fig. 2E, the presence of cell differentiation markers demonstrates differentiation of air- and WCS-exposed PBEC in our model. WCS exposure enhanced the number of LC3B^+^ puncta in ALI-PBEC (Fig. 2E), which was most pronounced in basal cells and goblet cells. Furthermore, the pronounced induction of autophagy markers in acute WCS models was largely absent in the chronic WCS-exposed ALI-PBEC cultures. Moreover, even a decreased ratio of LC3BII/I was observed 24 h after the last exposure, which was recovered after WCS cessation (Fig. 2F-H).

Taken together, acute WCS exposure of well-differentiated ALI-PBEC (representing an intact epithelial layer) resulted in an elevated abundance of autophagy markers associated with accumulation of autophagosomes and expression of ubiquitin-binding autophagic adaptors localized in both basal and luminal cells. Similar findings were made in undifferentiated S-PBEC exposed to CSE or WCS (representing damaged epithelium). Importantly, these changes were reversible as they were no longer observed after cessation in our chronic ALI-PBEC WCS exposure model.

### Modulation of mitophagy-specific markers in response to CS exposure

Because we observed a potent increase in the abundance of general autophagy markers in response to WCS exposure, and because these proteins are also essential for mitochondrial-specific autophagy (mitophagy), we next investigated whether the specific receptor- and ubiquitin-mediated mitophagy pathways involved in the removal of damaged or dysfunctional mitochondria were also affected by WCS exposure.

**Fig. 3.**
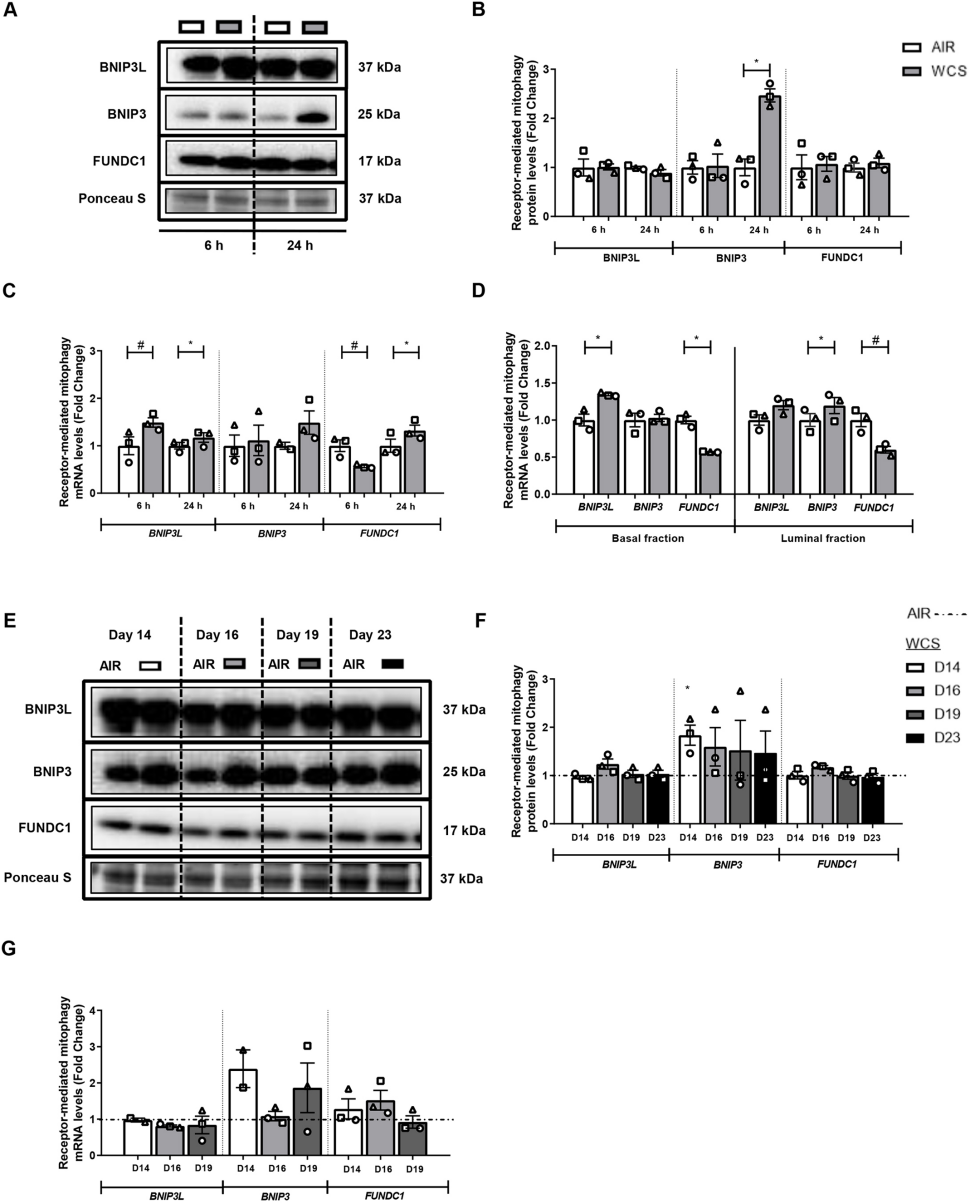
**WCS-induced changes in protein and mRNA levels of constituents involved in the receptor-mediated mitophagy machinery in ALI-PBEC.** After 2 weeks of differentiation, ALI-PBEC were exposed to fresh air or WCS from one 3R4F cigarette (University of Kentucky, 2 mg), and whole-cell lysates were harvested after 6 h and 24 h, and the basal and luminal fractions were harvested only at 6 h post-exposure (*n*=2-3 donors/group). (A-D) Protein (A,B) as well as mRNA (C,D) levels of constituents involved in receptor-mediated mitophagy – BNIP3L, BNIP3, FUNDC1 – were analyzed in whole-cell lysates or basal/luminal cell fractions post-exposure. Data are presented as mean fold change compared to control (air)±s.e.m. Independent donors are represented by open circles, triangles or squares. Statistical differences between WCS and air were tested using a two-tailed paired parametric *t*-test (^#^*P*<0.1 and *P<0.05). ALI-PBEC were 1× daily exposed to fresh air or WCS from one 3R4F cigarette (University of Kentucky, 2 mg) during differentiation for 14 days, followed by a cessation period of up to 10 days. Cells were harvested on Day 14 (24 h after the last exposure), 16, 19 and 23 (*n*=2-3 donors/group). (E-G) Protein (E,F) and mRNA (G) levels of receptor-mediated mitophagy regulators BNIP3L, BNIP3 and FUNDC1 were analyzed in whole-cell lysates. Representative western blots, including representative parts of the Ponceau S staining, are shown. Data are presented as mean fold change compared to control (air or WCS Day 14)±s.e.m. Independent donors are represented by open circles, triangles or squares. Statistical differences between WCS and air after smoking cessation in ALI-PBEC on each day were tested using a two-tailed paired parametric *t*-test (e.g. WCS Day 14 versus air). Comparison of various groups to test the difference between WCS Day 16, 19, 23 and Day 14 in WCS chronic smoking cessation experiments was conducted using one-way ANOVA followed by Sidak's post-hoc test for multiple comparisons, and, in the case of missing values, mixed-effects modeling was performed. Statistical significance is indicated as *P<0.05 compared to control (air or WCS Day 14).

As illustrated in Fig. 3, protein levels of BNIP3 and mRNA levels of *BNIP3L* and *FUNDC1* were significantly increased 24 h after acute WCS exposure in differentiated ALI-PBEC (Fig. 3A-C). In contrast, we observed a transient decrease in mRNA levels of *FUNDC1* at 6 h post-WCS exposure in these cultures (Fig. 3C). To study the impact of acute WCS exposure on regulators of mitophagy in specific epithelial cell types, we also investigated transcript levels of those regulators in the basal and luminal fractions of these differentiated ALI-PBEC cultures at 6 h post-WCS exposure. In line with the whole-cell lysate data, increased *BNIP3*(*L*) and decreased *FUNDC1* mRNA expression were observed in both fractions (Fig. 3D). The chronic WCS exposure model revealed that increases in BNIP3 protein levels in response to WCS were persistent 24 h after chronic WCS exposure, whereas changes in the transcript abundance of other regulators of mitophagy were more transient in nature (Fig. 3E-G). Although CSE exposure of undifferentiated S-PBEC yielded similar results as in ALI-PBEC cultures (i.e. increased BNIP3 protein), WCS exposure of these cells revealed only significantly decreased *BNIP3* mRNA levels (Fig. S4).

**Fig. 4.**
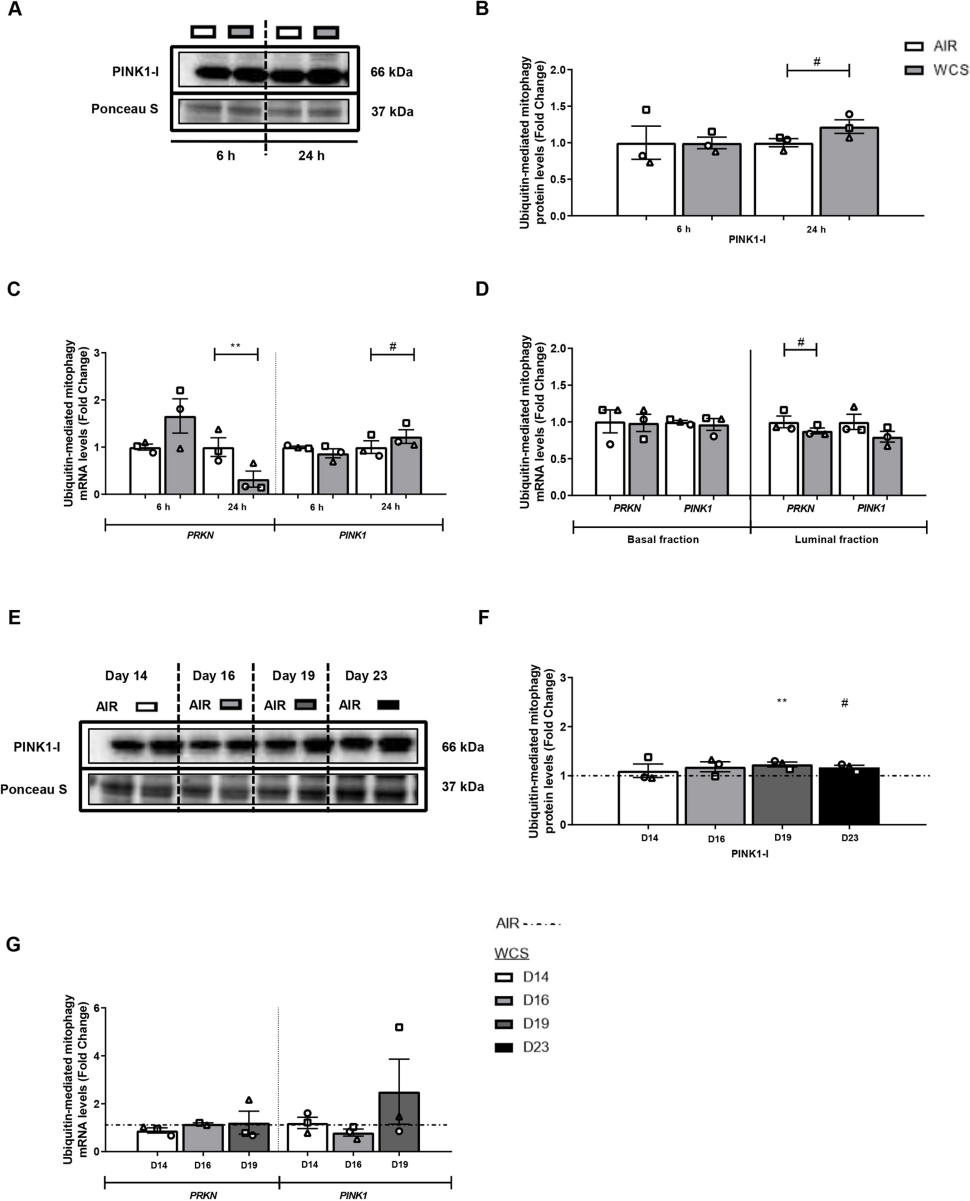
**Alterations in the abundance of constituents associated with ubiquitin-mediated mitophagy in ALI-PBEC.** After 2 weeks of differentiation, ALI-PBEC were exposed to fresh air or WCS from one 3R4F cigarette (University of Kentucky, 2 mg), and whole-cell lysates were harvested after 6 h and 24 h, and the basal and luminal fractions were harvested only at 6 h post-exposure (*n*=3 donors/group). (A-D) Protein (A,B) as well as mRNA (C,D) levels of constituents involved in ubiquitin-mediated mitophagy, PRKN and PINK1, were analyzed in whole-cell lysates or basal/luminal cell fractions post-exposure. Data are presented as mean fold change compared to control (air)±s.e.m. Independent donors are represented by open circles, triangles or squares. Statistical differences between WCS and air were tested using a two-tailed paired parametric *t*-test (^#^*P*<0.1 and **P<0.01). ALI-PBEC were 1× daily exposed to fresh air or WCS from one 3R4F cigarette (University of Kentucky, 2 mg) during differentiation for 14 days, followed by a cessation period of up to 10 days. Cells were harvested on Day 14 (24 h after the last exposure), 16, 19 and 23 (*n*=2-3 donors/group). (E-G) Protein (E,F) and mRNA (G) levels of mitophagy regulators PRKN and PINK1 were analyzed in whole-cell lysates. Western blot analysis revealed one distinct band for PINK1 protein corresponding with the expected molecular mass for PINK1-I (66 kDa). Representative western blots, including representative parts of the Ponceau S staining, are shown. Data are presented as mean fold change compared to control (air or WCS Day 14)±s.e.m. Independent donors are represented by open circles, triangles or squares. Statistical differences between WCS and air after smoking cessation in ALI-PBEC on each day were tested using a two-tailed paired parametric *t*-test (e.g. WCS Day 14 versus air). Comparison of various groups to test the difference between WCS Day 16, 19, 23 and Day 14 in WCS chronic smoking cessation experiments was conducted using one-way ANOVA followed by Sidak's post-hoc test for multiple comparisons, and, in the case of missing values, mixed-effects modeling was performed. Statistical significance is indicated as ^#^*P*<0.1 and **P<0.01 compared to control (air or WCS Day 14).

Because the abundance of the ubiquitin-binding autophagic adaptor SQSTM1 was increased in response to acute WCS exposure (Fig. 2), we further investigated the expression levels of ubiquitin-dependent mitophagy regulators PINK1 and PRKN. In general, PRKN abundance was significantly decreased in response to acute WCS exposure in ALI-PBEC and upon CSE treatment in S-PBEC, whereas PINK1 protein and transcript levels showed a trend to increase in acute WCS-exposed ALI-PBEC as well as upon smoking cessation (Fig. 4; Fig. S5).

In conclusion, these results indicate that WCS, as well as CSE, stimulation specifically affects the regulation of receptor-mediated mitophagy, which was consistently observed in all models and, at least to some degree, persists upon chronic WCS exposure and smoking cessation.

### WCS-induced alterations in the molecular machinery controlling mitochondrial biogenesis

**Fig. 5.**
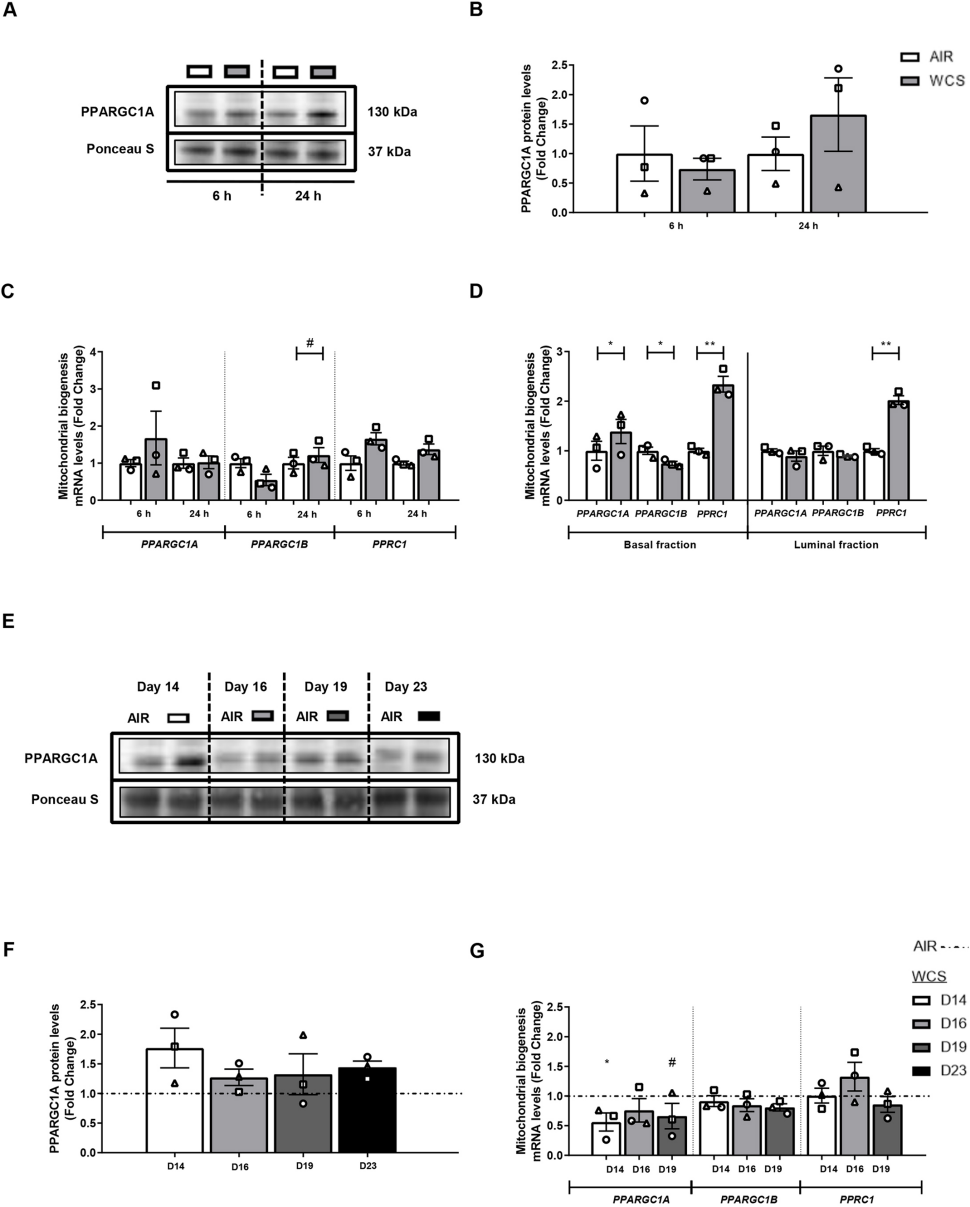
**Changes in transcription factors associated with mitochondrial biogenesis in response to WCS exposure in ALI-PBEC.** After 2 weeks of differentiation, ALI-PBEC were exposed to fresh air or WCS from one 3R4F cigarette (University of Kentucky, 2 mg), and whole-cell lysates were harvested after 6 h and 24 h, and the basal and luminal fractions were harvested only at 6 h post-exposure (*n*=3 donors/group). (A-D) Protein (A,B) and mRNA (C,D) levels of transcriptional co-activators of the PPARGC1 network, i.e. PPARGC1A, PPARGC1B and PPRC1, were analyzed in whole-cell lysates or basal/luminal cell fractions post-exposure. Data are presented as mean fold change compared to control (air)±s.e.m. Independent donors are represented by open circles, triangles or squares. Statistical differences between WCS versus air were tested using a two-tailed paired parametric *t*-test (^#^*P*<0.1, *P<0.05 and **P<0.01). ALI-PBEC were 1× daily exposed to fresh air or WCS from one 3R4F cigarette (University of Kentucky, 2 mg) during differentiation for 14 days, followed by a cessation period of up to 10 days. Cells were harvested on Day 14 (24 h after the last exposure), 16, 19 and 23 (*n*=3 donors/group). (E-G) Protein (E,F) and mRNA levels (G) of indices involved in the PPARGC1 network are depicted. Representative western blots, including representative parts of the Ponceau S staining, are shown. Data are presented as mean fold change compared to control (air or WCS Day 14)±s.e.m. Independent donors are represented by open circles, triangles or squares. Statistical differences between WCS and air after smoking cessation in ALI-PBEC on each day were tested using a two-tailed paired parametric *t*-test (e.g. WCS Day 14 versus air). Comparison of various groups to test the difference between WCS Day 16, 19, 23 and Day 14 in WCS chronic smoking cessation experiments was conducted using one-way ANOVA followed by Sidak's post-hoc test for multiple comparisons, and, in the case of missing values, mixed-effects modeling was performed. Statistical significance is indicated as ^#^*P*<0.1 and *P<0.05 compared to control (air or WCS Day 14).

As both mitophagy and mitochondrial biogenesis play an essential role in maintaining mitochondrial homeostasis, we next investigated the impact of CS on the abundance of constituents involved in the genesis of mitochondria. First, in ALI-PBEC, we evaluated whether acute WCS exposure affected the abundance of transcriptional co-activators of the PPARGC1 network, a critical signaling cascade involved in the regulation of mitochondrial biogenesis and mitochondrial energy metabolism (Scarpulla, 2011). No significant changes were observed in protein and transcript levels of most investigated transcriptional co-activators of the PPARGC1 network (i.e. PPARGC1A and PPARGGC1B) in whole-cell lysates in all models (Fig. 5; Fig. S6). However, elevated peroxisome proliferator-activated receptor gamma coactivator-related protein 1 (*PPRC1*) mRNA levels were observed in response to acute WCS exposure in both basal and luminal fractions of WCS-exposed ALI-PBEC (Fig. 5D). Similar responses, although not all statistically significant, likely due to interdonor variation, were observed in whole-cell lysates after WCS exposure of both differentiated ALI-PBEC and undifferentiated S-PBEC (Fig. 5C; Fig. S6C). Remarkably, *PPARGC1B* mRNA levels showed a transient response to acute WCS exposure, whereas we observed a decline in response to CSE in S-PBEC (Fig. 5C,D; Fig. S6C). In contrast to the response to acute WCS exposure, *PPARGC1A* mRNA levels were significantly decreased upon chronic WCS exposure (Fig. 5G).

**Fig. 6.**
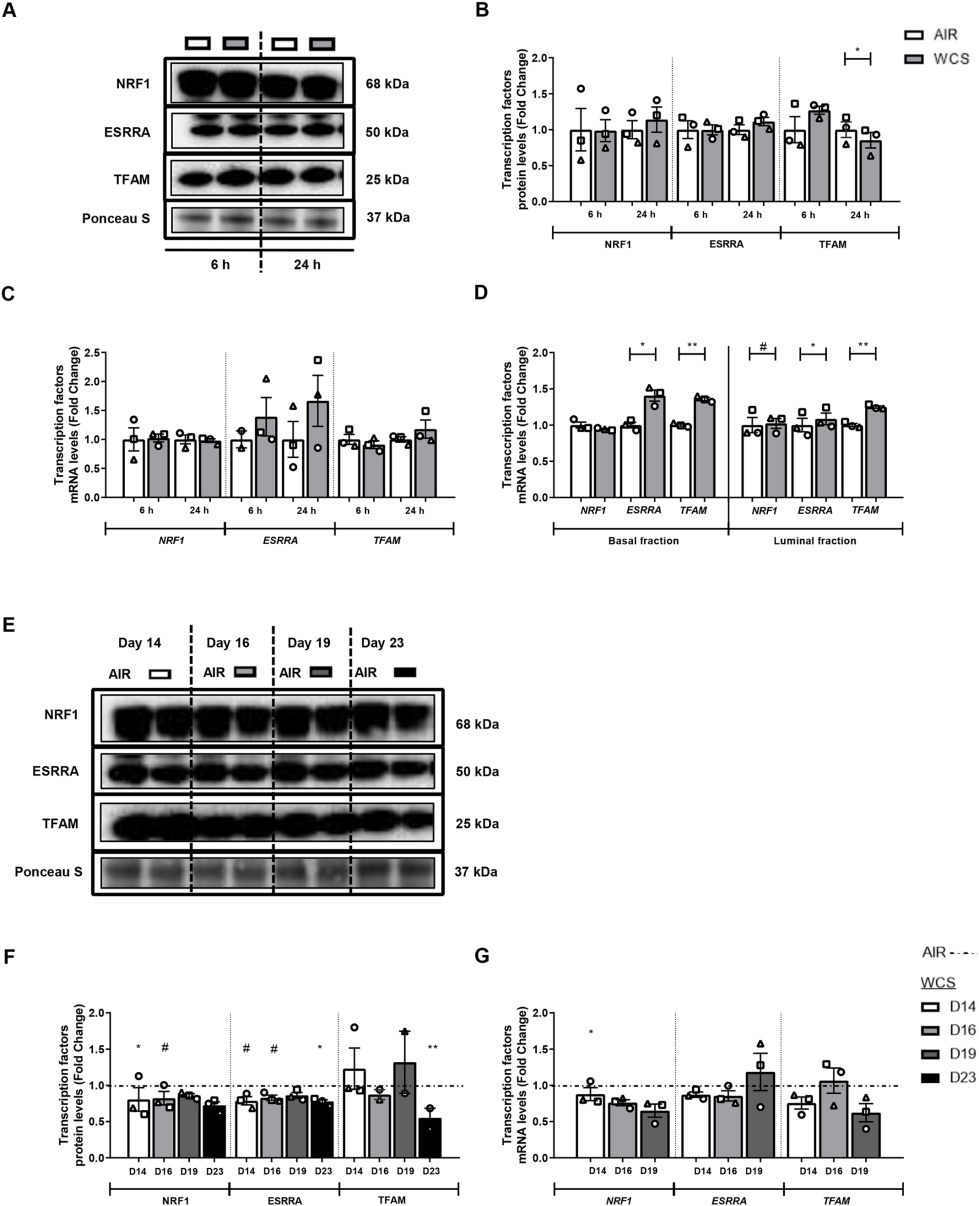
**Alterations in the abundance of PPARGC1-coactivated transcription regulators in WCS-exposed ALI-PBEC.** After 2 weeks of differentiation, ALI-PBEC were exposed to fresh air or WCS from one 3R4F cigarette (University of Kentucky, 2 mg), and whole-cell lysates were harvested after 6 h and 24 h, and the basal and luminal fractions were harvested only at 6 h post-exposure (*n*=2-3 donors/group). (A-D) Protein (A,B) as well as mRNA (C,D) levels of PPARGC1-coactivated transcription regulators: NRF1, ESRRA and TFAM were analyzed in whole-cell lysates or basal/luminal cell fractions post-exposure. Data are presented as mean fold change compared to control (air)±s.e.m. Independent donors are represented by open circles, triangles or squares. Statistical differences between WCS and air were tested using a two-tailed paired parametric *t*-test (^#^*P*<0.1, *P<0.05 and **P<0.01). ALI-PBEC were 1× daily exposed to fresh air or WCS from one 3R4F cigarette (University of Kentucky, 2 mg) during differentiation for 14 days, followed by a cessation period of up to 10 days. Cells were harvested on Day 14 (24 h after the last exposure), 16, 19 and 23 (*n*=2-3 donors/group). (E-G) Protein (E,F) and transcript (G) levels of PPARGC1-coactivated transcription regulators are presented. Representative western blots, including representative parts of the Ponceau S staining, are shown. Data are presented as mean fold change compared to control (air or WCS Day 14)±s.e.m. Independent donors are represented by open circles, triangles or squares. Statistical differences between WCS and air after smoking cessation in ALI-PBEC on each day were tested using a two-tailed paired parametric *t*-test (e.g. WCS Day 14 versus air). Comparison of various groups to test the difference between WCS Day 16, 19, 23 and Day 14 in WCS chronic smoking cessation experiments was conducted using one-way ANOVA, followed by Sidak's post-hoc test for multiple comparisons, and, in the case of missing values, mixed-effects modeling was performed. Statistical significance is indicated as ^#^*P*<0.1, *P<0.05 and **P<0.01 compared to control (air or WCS Day 14).

Thereafter, we examined the impact of WCS exposure on abundance of transcription factors associated with the PPARGC1 network. Protein and transcript levels of PPARGC1-coactivated transcription factors, either transcription factor A, mitochondrial (TFAM), nuclear respiratory factor 1 (NRF1) or estrogen-related receptor alpha (ESRRA), were largely unaltered upon acute WCS exposure and slightly decreased upon chronic WCS exposure and cessation in differentiated ALI-PBEC cultures (Fig. 6). Interestingly though, in line with increased levels of *PPRC1* as described above, fractionation of basal and luminal fractions revealed increased mRNA levels of *ESRRA* and *TFAM* in response to WCS (Fig. 6D). Variable, but minor, changes of the abundance of indices involved in mitochondrial biogenesis were observed in response to WCS (increases) and CSE (decreases) in undifferentiated S-PBEC cultures (Fig. S7).

Collectively, these data show that expression of transcriptional co-activators involved in mitochondrial biogenesis was largely unaltered in whole-cell lysates, while increased expression was observed in specific cell fractions of acute WCS-exposed ALI-PBEC and in basal cells of WCS-exposed S-PBEC. Moreover, a different response was observed in the other *in vitro* CS exposure models. However, the observed increases, in particular in PPRC1, and also alterations in the levels of some transcription factors associated with these co-activator molecules following CS exposure, may suggest a compensatory cellular response to induce mitochondrial biogenesis.

### Changes in the regulation of mitochondrial fusion and fission in response to WCS exposure

Because of the observed changes in these mitochondrial quality control mechanisms in the different models and the fact that these processes require mitochondrial fusion and fission, we examined the impact of WCS exposure on key molecules involved in the regulation of mitochondrial dynamics. Acute WCS treatment resulted in upregulation of the mitochondrial fusion marker mitofusin 1 (*MFN1*) in ALI-PBEC (Fig. S8A-E). However, CSE or WCS exposure had no pronounced impact on the regulation of fission or fusion markers in S-PBEC (Fig. S9). Also, no pronounced alterations were observed in the abundance of mitochondrial dynamic regulators upon chronic WCS exposure and the smoking cessation period (Fig. S8F-H).

### Disruption of the metabolic phenotype upon WCS exposure

We next investigated whether CS-induced changes in the regulation of mitochondrial biogenesis and mitophagy were accompanied by changes in mitochondrial content and mitochondrial metabolic pathways. To this end, we investigated the effects of WCS exposure on the activity of critical metabolic enzymes and abundance of proteins involved in mitochondrial metabolic pathways.

Acute WCS exposure of differentiated ALI-PBEC cultures, as well as CSE exposure of undifferentiated S-PBEC, did not affect mitochondrial content, as assessed by mitochondrial DNA (mtDNA) copy number (Fig. S10A, Fig. S11A). Also, protein and mRNA levels of nuclear-encoded and mitochondrial-encoded proteins and genes of electron transport chain (ETC) subunits were unaltered after acute or chronic exposure of ALI-PBEC to WCS (Fig. S10B-H). Some minor, but variable, alterations were found in transcript and protein levels of analyzed subunits of ETC complexes in undifferentiated S-PBEC cultures exposed to CSE or WCS (Fig. S11B-D). Although we did observe some slight alterations in the levels of ETC subunits in the different models, in general, our data indicate no marked changes in indices of mitochondrial content in all models after acute or chronic exposure to CS.

We also examined the impact of CS exposure on the activity and expression of constituents of the fatty acid β-oxidation and tricarboxylic acid cycle. Besides downregulated hydroxyacyl-coenzyme A dehydrogenase (HADH) enzyme activity in acute WCS-exposed ALI-PBEC, we also observed increased citrate synthase activity in CSE-treated S-PBEC (Fig. S12A,E). Similar changes at mRNA level were found in the basal fraction of WCS-exposed ALI-PBEC, while conflicting, but transient, gene expression was found in S-PBEC CS models (Fig. S12B-D,F).

**Fig. 7.**
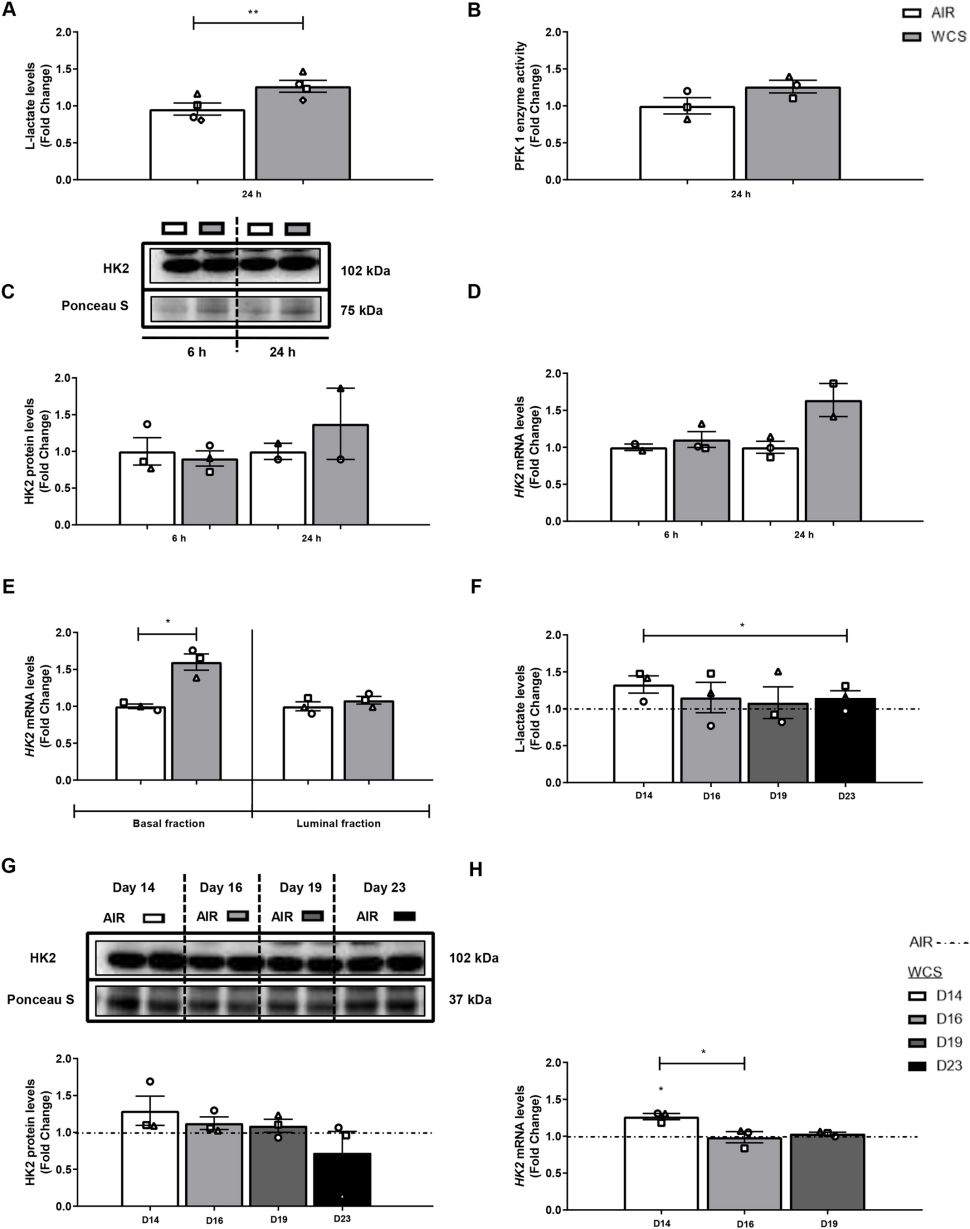
**WCS-induced shift to anaerobic glycolysis in ALI-PBEC.** After 2 weeks of differentiation, ALI-PBEC were exposed to fresh air or WCS from one 3R4F cigarette (University of Kentucky, 2 mg), and basal medium was collected after 24 h, and whole-cell lysates were harvested after 6 h and 24 h, and the basal and luminal fractions were harvested only at 6 h post-exposure (*n*=2-4 donors/group). (A-E) L-lactate in basal medium (*n*=4 donors/group) (A), PFK1 activity (*n*=3 donors/group) (B), and HK2 protein (C) and mRNA (D,E) (*n*=2-3 donors/group) levels were analyzed in whole-cell lysates or basal/luminal cell fractions post-exposure. Data are presented as mean fold change compared to control (air)±s.e.m. Independent donors are represented by open circles, triangles, squares or diamonds. Statistical differences between WCS and air were tested using a two-tailed paired parametric *t*-test (*P<0.05 and **P<0.01). ALI-PBEC were 1× daily exposed to fresh air or WCS from one 3R4F cigarette (University of Kentucky, 2 mg) during differentiation for 14 days, followed by a cessation period of up to 10 days. Basal medium and cells were harvested on Day 14 (24 h after the last exposure), 16, 19 and 23 (*n*=3 donors/group). (F-H) L-lactate (F), and HK2 protein (G) and transcript (H) levels are shown. Representative western blots, including representative parts of the Ponceau S staining, are shown. Data are presented as mean fold change compared to control (air or WCS Day 14)±s.e.m. Independent donors are represented by open circles, triangles or squares. Statistical differences between WCS and air after smoking cessation in ALI-PBEC on each day were tested using a two-tailed paired parametric *t*-test (e.g. WCS Day 14 versus air). Comparison of various groups to test the difference between WCS Day 16, 19, 23 and Day 14 in WCS chronic smoking cessation experiments was conducted using one-way ANOVA followed by Sidak's post-hoc test for multiple comparisons, and, in the case of missing values, mixed-effects modeling was performed. Statistical significance is indicated as *P<0.05 compared to control (air or WCS Day 14).

Finally, we explored whether CS exposure may affect the metabolic program in PBEC. First, lactate production was measured in the basal medium of WCS-exposed ALI-PBEC. We observed an increase in L-lactate levels after acute and chronic WCS exposure of differentiated ALI-PBEC cultures compared to air control, which persisted after WCS cessation, indicating a shift to a glycolytic metabolism (Fig. 7A,F). In line with this, mRNA abundance of hexokinase 2 (HK2), the enzyme responsible for the first step in glycolysis, was found to be elevated in acute and chronic WCS-exposed ALI-PBEC and in S-PBEC (Fig. 7E,H; Fig. S13E). Activity or expression levels of other markers of glycolysis [phosphofructokinase 1 (PFK1) enzyme activity and HK2 abundance] showed no significant differences in response to acute or chronic WCS-exposed ALI-PBEC (Fig. 7B-D,G). No significant differences were observed in L-lactate levels after acute WCS exposure in undifferentiated S-PBEC (Fig. S13A), or in activity of PFK1 and expression of HK2 in CSE-exposed S-PBEC (Fig. S13B-E).

Collectively, these results demonstrate that CS exposure does not affect mtDNA copy number or the abundance of constituents of the ETC, but do suggest a shift to a more glycolytic metabolism in fully differentiated PBEC cultures, but not in damaged epithelium.

## DISCUSSION

In our study, for the first time, we examined the impact of acute WCS and CSE exposure on the molecular mechanisms regulating mitochondrial content and function in multiple PBEC models, ranging from undifferentiated cells that were exposed to CSE in a submerged culture to well-differentiated cultures exposed to WCS in an ALI system. In addition to studying acute effects of exposure, we also evaluated the persistence of these effects following chronic WCS exposure during differentiation and potential recovery upon cessation of WCS exposure.

First and foremost, we observed a potent increase in the abundance of general autophagy proteins in differentiated cultures acutely exposed to WCS, in both basal and luminal cell fractions. Interestingly, these changes were largely absent in PBEC repeatedly exposed to WCS during differentiation. This autophagy response to acute smoke exposure was also observed in undifferentiated PBEC, both in response to acute WCS and CSE exposure. Also, mitophagy-related protein and transcript expression, specifically for those involved in receptor-mediated mitophagy, increased in response to smoke exposure in several of our models. Increases in the expression of proteins involved in receptor-mediated mitophagy were mainly observed in differentiated cultures, persisted after chronic exposure and remained elevated for the duration of the smoke cessation protocol. Analysis of markers of mitochondrial content and mitochondrial dynamics revealed no pronounced changes in response to WCS or CSE in any of our models. We did observe, however, that differentiated PBEC cultures acutely exposed to WCS displayed minor changes in the abundance of proteins involved in mitochondrial biogenesis, as well as a metabolic shift towards a more glycolytic phenotype. These findings are summarized in Table 1, which also shows several differences in the response of our different PBEC cultures to WCS or CSE. This highlights the importance of tailoring the *in vitro* model to the research question with regard to studying the impact of (chemical) exposure-induced mitochondrial abnormalities in the context of respiratory disease.

**Table 1.**
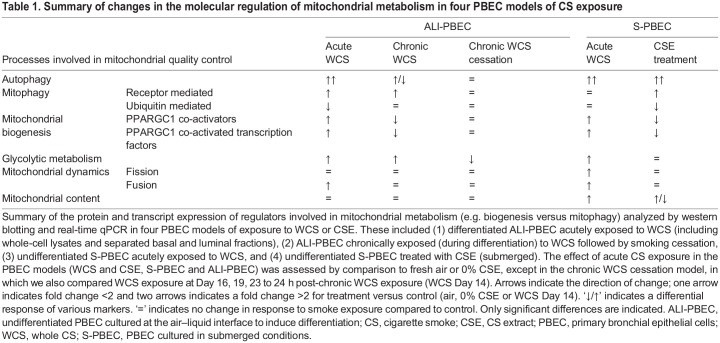
Summary of changes in the molecular regulation of mitochondrial metabolism in four PBEC models of CS exposure

We observed increases in both protein and transcript levels of key constituents of autophagy in luminal as well as basal fractions of differentiated cultures as well as in whole-cell lysates of non-differentiated cultures. Collectively, this implies that this is a robust response of different bronchial epithelial cell types to CS constituents and is reflected in both intact as well as damaged epithelium (both of which are present in the airways of COPD patients). These data are in line with previous findings in CS(E)-treated human airway epithelial cells (Chen et al., 2008; Ito et al., 2015; Park et al., 2019; Xu et al., 2019; Zhang et al., 2019a), in lung homogenates of (a majority of ex-smoking) COPD patients (Chen et al., 2008, 2010) and in CS-exposed mice (Chen et al., 2008, 2010; Mizumura et al., 2014). Furthermore, they provide further support for the hypothesis that imbalanced autophagy, and in particular excessive autophagy induction, contributes to the pathogenesis of COPD by resulting in programmed cell death of epithelial cells and subsequent development of pulmonary emphysema (Ornatowski et al., 2020).

Induction of autophagy likely results from (sub)cellular damage by oxidative components present in CS (Ornatowski et al., 2020; Yun et al., 2020). In line with this notion and with previous studies (Ito et al., 2015), we did observe modulation of cellular antioxidant systems in response to WCS in our models. Although a robust induction of autophagy markers was observed in our acute exposure models, chronic exposure to WCS resulted in a decreased ratio of LC3BII/I in differentiating PBEC, which recovered during smoke cessation. In line with this observation, a recent study showed low amounts of LC3B-positive cells in cultured PBEC from severe COPD patients, possibly associated with a decreased number of club cells (Malvin et al., 2019). As a persistent loss of the club cell marker SCGB1A1 has previously been demonstrated in our chronic WCS model (Amatngalim et al., 2018), the differential effects of chronic versus acute WCS exposure on autophagy markers and recovery upon cessation could potentially be explained by both oxidative stress and aberrant differentiation and/or loss of club cells in response to chronic smoke exposure (Amatngalim et al., 2018). These speculations on the relationship between autophagy and aberrant epithelial cell differentiation are supported by observations showing that autophagy inhibits ciliated cell differentiation (Li et al., 2019) and regulates mucin production by goblet cells in the airway epithelium (Zhou et al., 2016). Although blocking induction of autophagy (e.g. after smoke exposure) may improve epithelial function (Wen et al., 2019; Zhang et al., 2019a), autophagy may also serve a protective function by enabling adequate restoration of airway epithelial function after an insult (Li et al., 2017; Pampliega and Cuervo, 2016). Collectively, these studies indicate that CS-induced increases in autophagy contribute to aberrant epithelial differentiation upon smoke exposure.

Mitophagy is a form of targeted autophagy that can contribute to the clearance of damaged mitochondria that have been found following exposure to CS in multiple human and mice airway epithelial cell types in lung tissue as well as in PBEC cultures from COPD patients (Ahmad et al., 2015; Ballweg et al., 2014; Ito et al., 2015). In our study, we found an increased abundance of proteins involved in receptor-mediated mitophagy following both acute and chronic stimulation with WCS or CSE in different cell populations, which persisted during the cessation period. To the best of our knowledge, only a few studies have investigated the impact of CS on regulation of receptor-mediated mitophagy in airway epithelial cells. These studies reported an increase in receptor-mediated mitophagy proteins in response to smoke exposure both *in vivo* and *in vitro* (Wen et al., 2019; Zhang et al., 2019a).

Interestingly, it has been shown that cellular hypoxia-related signaling [i.e. hypoxia-inducible factor (HIF1α)] is activated by smoke exposure in these airway epithelial cell models (Daijo et al., 2016; Yasuo et al., 2011; Zhang et al., 2007). Moreover, it is known that hypoxia activates receptor-mediated mitophagy in various cell types (Bellot et al., 2009; Ishihara et al., 2013; Lee et al., 2011; Lv et al., 2017; Wang et al., 2018a; Wu et al., 2016), as HIF1α has been recognized as an upstream regulator of BNIP3(L) (Allen et al., 2013; Bellot et al., 2009; Wang et al., 2016). Therefore, it can be speculated that CS-induced hypoxia partly contributes to the observed CS-induced elevated receptor-mediated mitophagy. Moreover, indicative of a contribution of CS-induced activation of receptor-mediated mitophagy to COPD pathology, one study reported amelioration of COPD-like features in mice with genetically blocked receptor-mediated mitophagy (Wen et al., 2019). In contrast to the observed robust changes in receptor-mediated mitophagy in response to CS in our models, changes in mediators of ubiquitin-mediated mitophagy were less pronounced. In general, we observed that PINK1 protein and transcript levels tended to be increased in response to acute CS exposure, whereas PRKN levels decreased. This is in line with the literature, as CS-induced increases in PINK1 levels and decreased PRKN abundance have been described in several *in vitro*/*vivo* (airway) models (Ahmad et al., 2015; Ito et al., 2015; Kyung et al., 2018; Mizumura et al., 2014; Son et al., 2018; Wu et al., 2020) and in peripheral lung tissue and bronchial epithelial cells of (ex-smoking) COPD patients (Ahmad et al., 2015; Hoffmann et al., 2013; Ito et al., 2015; Mizumura et al., 2014). However, other studies reported no changes or even increases in the levels of these constituents of ubiquitin mitophagy in response to CS in various *in vitro* and *in vivo* models of exposure of cells of the airways to CS as well as in lungs of COPD patients (Ahmad et al., 2015; Ballweg et al., 2014; Hoffmann et al., 2013; Mizumura et al., 2014; Sundar et al., 2019; Wu et al., 2020).

In general, whether mitophagy serves a protective or detrimental role in CS-induced COPD development remains controversial. Indeed, previous studies have reported that knockdown/knockout of PINK1 or PRKN (Araya et al., 2019; Ito et al., 2015; Kyung et al., 2018; Mizumura et al., 2014), as well as overexpression of PRKN (Ahmad et al., 2015; Araya et al., 2019; Ito et al., 2015), protected against CSE-induced mitophagy or mitochondrial dysfunction in *in vitro* or *in vivo* models, highlighting the complexity of regulation of mitophagy. Seemingly discrepant findings regarding CS-induced mitophagy in the literature may stem from differences in dose and time of exposure of cells to CS constituents (mild versus severe CS stress) or from the dynamic (flux) nature of mitophagy. The fact that, in our study, the induction of receptor-mediated mitophagy was more pronounced than ubiquitin-mediated mitophagy and that *in vivo* models of smoke exposure (or COPD) often describe activation of this PINK1/PRKN pathway (Ahmad et al., 2015; Ito et al., 2015; Mizumura et al., 2014), may be related to the fact that our *in vitro* models lack the inflammatory cells that are present *in vivo* in smoke-induced COPD. An important consideration is that inflammation is linked to mitochondrial dysfunction (Kampf et al., 1999; López-Armada et al., 2013) and inflammatory mediators may activate the PINK1/PRKN pathway in epithelial cells (Liu et al., 2021). Further studies focusing on the molecular mechanisms of the pathway-specific regulation of mitophagy in epithelial cell types need to be conducted.

Besides clearance of damaged mitochondria by mitophagy, mitochondrial biogenesis is crucial in maintaining mitochondrial homeostasis. Interestingly, we observed that some proteins involved in mitochondrial biogenesis were increased in response to acute WCS exposure, while both CSE and chronic WCS exposure resulted in a decrease in these molecules. In line with our findings, previous studies also observed differential effects of short-term or long-term CS exposure on mitochondrial biogenesis. For example, whereas short-term CSE treatment of cultured human bronchial epithelial cells increased transcript abundance of the mitochondrial biogenesis-associated marker PPARGC1A (Vanella et al., 2017), PPARGC1A was non-detectable after long-term CSE exposure (Hoffmann et al., 2013). Similar findings were also observed in lung tissue from COPD patients at different disease stages, ranging from increased PPARGC1A abundance in mild or ex-smoking COPD patients to decreased abundance in moderate and severe COPD lung tissue (Hoffmann et al., 2013; Li et al., 2010). Collectively, increased mitochondrial biogenesis in acute WCS models might indicate an adaptive cellular response, whereas decreased mitochondrial biogenesis upon chronic WCS exposure might reflect an inability to compensate for these changes.

We did not find pronounced differences in the abundance of fusion and fission indices after CS exposure in our models. Previous studies have found that short- or long-term CSE exposure can induce changes in proteins regulating mitochondrial dynamics with effects on mitochondrial morphology (Ballweg et al., 2014; Mizumura et al., 2014). These effects were found to range from swollen, fragmented organelles to mitochondrial hyperfusion (Aravamudan et al., 2014; Ballweg et al., 2014; Hara et al., 2013; Kyung et al., 2018; Mizumura et al., 2014; Son et al., 2018; Song et al., 2017; Sundar et al., 2019), and the variability in CSE preparation (including type of cigarette used, concentration and generation of CSE) may explain the conflicting findings in *in vitro* studies. As we did not assess mitochondrial morphology in our study, no solid conclusions can be drawn about the impact of CS exposure in our models.

mtDNA damage and disturbed mitochondrial metabolism, including impaired respiratory capacity and abundance/activity of subunits of ETC complexes, have been described in multiple *in vitro* or *in vivo* (airway) models in response to CS(E) (Aghapour et al., 2020; Cloonan et al., 2016; Hoffmann et al., 2013; Mizumura et al., 2014; Park et al., 2019; Sundar et al., 2019; Wu et al., 2020). Importantly, several studies showed that CS-induced mitochondrial dysfunction contributes to altered epithelial function (Yang et al., 2021) as well as development of COPD (Cloonan et al., 2016). In our study, we did not observe large changes in mtDNA copy number or the abundance of oxidative phosphorylation complexes. As mtDNA copies vary per cell type, and only selected subunits of various complexes were analyzed, additional studies are required. We cannot exclude the possibility that increased mitochondrial biogenesis may have compensated for potential loss of mitochondria in our models.

In line with prior evidence reporting CS-induced metabolic reprogramming of airway epithelial cells (Agarwal et al., 2014; Solanki et al., 2018), mouse lungs (Agarwal et al., 2012) or in COPD (Agarwal et al., 2019; Kao et al., 2012; Pastor et al., 2013; Tu et al., 2014), we observed increased lactate production in our models in response to WCS, which persisted during chronic WCS exposure and cessation. Although we did not assess mitochondrial function by respirometry or complex activity analysis, and therefore cannot decisively conclude about the impact of CS on mitochondrial function, the fact that lactate increases does suggest abnormalities in mitochondrial oxidative metabolism. Moreover, metabolic reprogramming is suggested to be a driver of CS-induced inflammatory lung diseases (Li et al., 2021). Furthermore, it has been reported that lactate, here found to be increased after smoke exposure as a result of smoke-induced deregulation of cellular metabolism, directly binds to transmembrane domain of mitochondrial antiviral-signaling protein (MAVS) and thus prevents MAVS aggregation. This is an important step in antiviral signaling, and it was reported that lactate inhibits type I interferon production, impairs antiviral responses and enhances viral replication (Zhang et al., 2019b). Because it is well established that cigarette smoking increases susceptibility to viral infections (Jiang et al., 2020), which was replicated in various *in vitro* epithelial culture models (including our WCS for rhinovirus infection) (Duffney et al., 2018; Eddleston et al., 2011; Wang et al., 2019), these data suggest that increased lactate production upon WCS exposure may contribute to CS-induced increases in epithelial susceptibility to viral infections.

To better understand the impact of CS on mitochondrial quality control processes in simple and advanced *in vitro* PBEC models, we included four models that cover variation in type and duration of CS exposure, epithelial cell types and read-out parameters. Obviously, the different exposure scenarios influence the results because, during WCS (that includes volatiles) exposure, the cells are directly and shortly exposed, whereas via incubating with CSE (only dissolved, aqueous components) in the medium, the contact time with CS components will be longer. Although a similar response in these two exposure models can be explained by the impact of aqueous CS components (present in both CS and CSE), a conflicting response could be due to the continuous exposure of CSE (e.g. receptor-mediated mitophagy) or additional volatiles present in WCS. However, in general, the non-standardized methods of WCS or CSE preparation, e.g. variability in type of cigarette, concentration determination and generation (often in-house-developed systems), make it difficult to compare results of different smoke exposure studies.

**Fig. 8.**
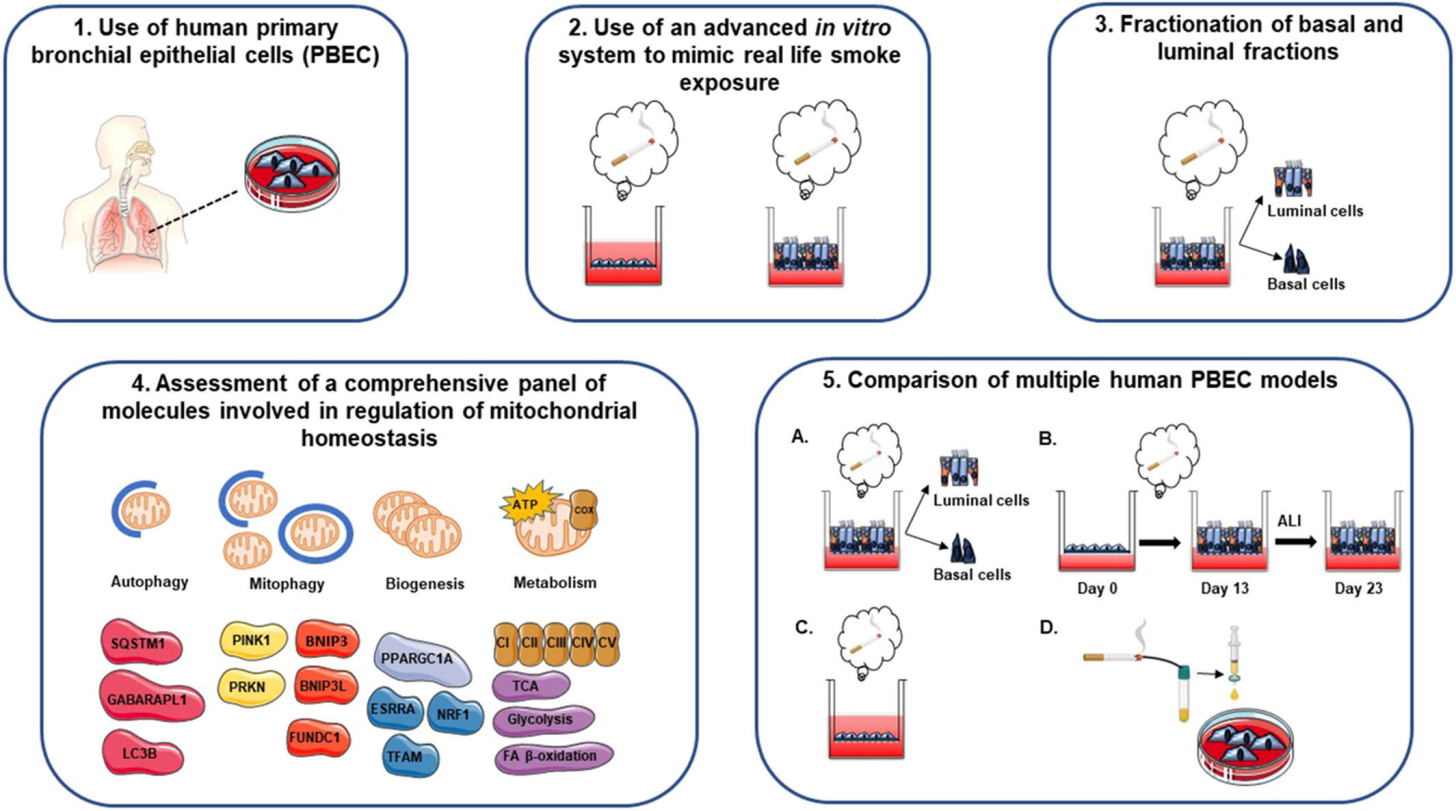
**Overview of experimental approach.** Schematic representation of the novelty and strengths of the study. (1) Use of human primary bronchial epithelial cells (PBEC): human PBEC (differentiated or undifferentiated) from multiple donors [non-chronic obstructive pulmonary disease (COPD) patients] were used to assess CS-induced changes in mitochondrial homeostasis. (2) Use of an advanced *in vitro* system to mimic real-life smoke exposure: fully differentiated cultures of PBEC were directly and shortly exposed to WCS in an air–liquid interface (ALI) system. (3) Fractionation of basal and luminal fractions: changes in the regulation of mitochondrial homeostasis in basal and luminal fractions of WCS-exposed differentiated PBEC were investigated. (4) Assessment of a comprehensive panel of molecules involved in regulation of mitochondrial homeostasis: a comprehensive panel of markers involved in all important processes associated with the regulation of mitochondrial metabolism was investigated in our *in vitro* CS exposure models of PBEC. (5) Comparison of multiple human PBEC models (A-D): studying a subset of molecules involved in mitochondrial metabolism in four relevant *in vitro* CS exposure models of PBEC allows important comparisons not only between our four different models, but also with data present in literature.

The strength of our study is the investigation, for the first time, of a comprehensive panel of markers involved in the regulation of mitochondrial metabolism in various relevant CS exposure *in vitro* primary human airway epithelial cell models. The novelty of our findings is multifaceted (Fig. 8). First, the use of human PBEC from multiple donors (Table 2) to assess CS-induced changes in mitochondrial homeostasis is a strength as many studies in this area use immortalized or tumor lines or rodent exposure studies. Second, we exposed fully differentiated cultures of PBEC directly and shortly to WCS (gas and particulate phase, short- and long-lived CS constituents) in an ALI system, which adequately mimics the real-life exposure. Reports using this model and exposure system are very limited (likely due to the complexity of the culture and exposure system), and most studies using primary cells employed undifferentiated submerged cultures that were exposed to CSE rather than WCS. Moreover, the combination of these various models (CSE and WCS, undifferentiated S-PBEC and differentiated ALI-PBEC) adds important novelty and insight to the field. Third, we analyzed changes in the regulation of mitochondrial homeostasis in both luminal and basal cell fractions of the differentiated pseudostratified epithelium, which has not previously been reported. Fourth, whereas studies usually examined a selection of markers related to one or two mitochondrial processes, we here investigated for the first time a comprehensive panel of markers involved in all important processes associated with the regulation of mitochondrial metabolism in our *in vitro* CS exposure models of PBEC. Lastly, studying this subset of molecules involved in mitochondrial metabolism in four relevant *in vitro* CS exposure models of PBEC allows important comparisons not only between our four different models, but also with data present in the literature. The relevance of our findings is enhanced by using PBEC cultured in both (un)differentiated submerged and ALI conditions and comparison of different type and duration of CS exposure.

**Table 2.**
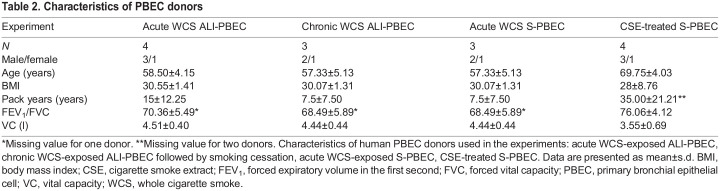
Characteristics of PBEC donors

The differentiated epithelial cultures used in the present study mimic an intact pseudostratified epithelium relevant to study responses to inhalation toxicants of an intact epithelial layer, while the undifferentiated PBEC model represents a basal cell-like structure relevant to study responses of a damaged, partly denuded epithelial layer. Moreover, chronic WCS exposure of a differentiating epithelial layer followed by cessation of exposure, provided the opportunity to investigate repair. Strikingly, we observed abnormalities at the level of mitochondrial quality control mechanisms in all models, suggestive of a role for CS-induced mitochondrial dysfunction in different areas of the bronchial epithelium relevant for COPD. The different responses that we observed highlight the importance of making considered choices for (un)differentiated cell models, type of CS exposure and duration of smoking in future respiratory inhalation (toxicology) studies tailored to the research question.

Inevitably, our study obviously also has several limitations. First, a limited number of donors was investigated. Second, although our short-term CS exposure models may mimic changes in airway epithelial cells from chronic smokers, including autophagy, differences between molecular changes in response to acute versus chronic smoke exposure or with changes in lung homogenates from COPD patients were observed, as illustrated by the findings on mitochondrial biogenesis or ubiquitin-mediated mitophagy. Furthermore, as mitochondrial biogenesis, mitophagy and mitochondrial dynamics are dynamic processes, the fact that our analyses only represent a snapshot of these processes is a limitation. Because our current research aimed to compare CS-induced alterations in the molecular mechanisms underlying mitochondrial function between four different models, we did not focus on their contribution to CS-induced changes in epithelial function related to, for instance, repair, differentiation and remodeling, as well as, for example, epithelial barrier function, antimicrobial responses, cilia beating and mucus production. However, the impact of acute and chronic CS exposure on the abovementioned aspects of epithelial function has previously been reported by us in the models used in the present study (Amatngalim et al., 2016, 2018, 2017, 2015; Luppi et al., 2005; Wang et al., 2019) and by other research groups (Aghapour et al., 2018; Cao et al., 2018; Gindele et al., 2020; Heijink et al., 2012; Tatsuta et al., 2019). In addition, direct associations between mitochondrial dysfunction, deregulated autophagy and lactate production and epithelial function have been reported previously (Cloonan et al., 2016; Fujita et al., 2015; Li et al., 2019; Wen et al., 2019; Yang et al., 2021; Zhang et al., 2019a; Zhou et al., 2016). Whereas we only separated basal and luminal cells in our differentiated ALI-PBEC model that includes several epithelial cell types (Amatngalim et al., 2018), additional research should evaluate the impact of CS exposure in individual luminal cell types, as well as in alveolar cells, as those are also markedly affected in COPD. Furthermore, further research is needed to delineate the effects of CS exposure on mitochondrial quality control processes, using advanced state-of-the-art *in vitro* models and more realistic WCS exposure methods, including varying puff-topography regimes reflecting human smoking behavior.

Our model is limited to (differentiated) epithelial cells and did not include other cell types such as immune cells. However, our observations are supported by findings in lung tissue of COPD patients showing mitochondrial alterations and increased autophagy. Moreover, other studies reported similar molecular changes of mitochondrial metabolism in *in vivo* smoke exposure models and have demonstrated that these are not only associated with, but also causally related to, functional and structural abnormalities in the bronchial and alveolar compartment reminiscent of COPD lung/airway pathology (Chen et al., 2008; Cloonan et al., 2016; Mizumura et al., 2014). Future studies using more complex models such as co-culture (Iskandar et al., 2015), lung on chips (Bennet et al., 2021) or *ex vivo* precision-cut lung slices (Donovan et al., 2016) to investigate the individual molecular changes at global, single-cell and tissue levels, have the potential to overcome some of these limitations. We have previously reported the feasibility of such an approach by showing that macrophages support epithelial repair in our WCS exposure model using co-cultures of epithelial cells and monocyte-derived macrophages (van Riet et al., 2020). Moreover, although our study has a targeted approach, future studies could focus on a more ‘global’ multi-omics approach to investigate the impact of smoke exposure on mitochondrial metabolism. Other studies have used such approaches; for example, metabolomics has been used to elucidate the role of smoke-induced metabolic reprogramming in the pathogenesis of lung diseases (Li et al., 2021), lipidomics was used by us to show that polyunsaturated fatty acid metabolism is affected by exposure of ALI-PBEC to WCS and altered in sputum from COPD patients compared to controls (van der Does et al., 2019), and transcriptomics analysis of CS-exposed ALI cultures reported critical molecular pathways involved in aberrant tissue remodeling and lung disease-associated pathways (Xiong et al., 2021). Interestingly, a multi-omics approach has also been applied in repeated CS-exposed co-cultures of bronchial tissue, revealing alterations in molecular pathways involved in airway diseases (Ishikawa et al., 2019). However, these studies did not compare the different culture or exposure models used in the present study.

In conclusion, in this study, differences were observed in the regulation of mitochondrial metabolic processes in the four investigated models reflecting damaged, intact, differentiating or repairing airway epithelium, and multiple CS exposure regimes. The results highlight the robust model-independent impact of CS exposure on the abundance of key molecules involved in autophagy and receptor-mediated mitophagy. These alterations were, at least in part although less pronounced, recapitulated after chronic exposure of differentiating epithelial cells to CS. Differences observed in the regulation of mitochondrial metabolic processes, such as mitochondrial biogenesis, in the investigated models, reflecting various cell types of the epithelium and CS exposure regimes, supports the importance of tailoring the experimental model to the research question.

## MATERIALS AND METHODS

### PBEC isolation and expansion

PBEC of four non-COPD donors for the CSE exposure experiments of undifferentiated PBEC were provided by the Primary Lung Culture (PLUC) facility at Maastricht University Medical Center+ (Maastricht, The Netherlands) and PBEC of four non-COPD donors for the WCS exposure experiments of (un-)differentiated PBEC were obtained from Leiden University Medical Center (Leiden, The Netherlands). Approval of the use of PBEC for research provided by the PLUC facility was confirmed by the scientific board of the Maastricht Pathology Tissue Collection (MPTC) under code MPTC2010-019 and the local Medical Ethic Committee code 2017-0087. PBEC were isolated, expanded and differentiated as previously described (Amatngalim et al., 2015; Schrumpf et al., 2020; van Wetering et al., 2000, 2007). Use of such lung tissue that became available for research within the framework of patient care at MUMC+ and LUMC was in line with the ‘Human Tissue and Medical Research: Code of conduct for responsible use’ (2011) (www.federa.org), which describes the opt-out system for coded anonymous further use of patient data and tissue collection, storage and further use. Characteristics of the PBEC donors are shown in Table 2.

In brief, PBEC were isolated from tumor-free resected bronchus rings obtained from lung cancer patients undergoing resection surgery at MUMC+ or LUMC. After isolation, PBEC were seeded and expanded in keratinocyte serum-free medium (Life Technologies Europe B.V., The Netherlands) containing 0.2 ng/ml epidermal growth factor (Gibco, USA), 25 µg/ml bovine pituitary extract (Gibco or Life Technologies Europe B.V.), 1 µM isoproterenol (Sigma-Aldrich, USA) and 100 µg/ml primocin (InvivoGen, The Netherlands) or Mycozap (0.2%) (Lonza, USA) on six-well plates (Corning Costar, USA) coated with 5 μg/ml or 10 μg/ml human fibronectin (Promocell, Germany or Sigma-Aldrich), 30 μg/ml PureCol (Advanced BioMatrix, USA) and 10 μg/ml bovine serum albumin (BSA; Fraction V; Sigma-Aldrich or Thermo Fisher Scientific, USA) diluted in Hank's balanced salt solution (HBSS; no calcium, no magnesium, no Phenol Red) (Gibco) or phosphate-buffered saline (PBS; Fresenius Kabi, The Netherlands). When cells reached confluence, PBEC (mycoplasma-free) were harvested by trypsinization and frozen in liquid nitrogen until use.

### Submerged PBEC culture and CSE treatment

PBEC of four non-COPD donors (MUMC+) were thawed (5×10^5^ cells, passage 1), seeded and further expanded in supplemented keratinocyte serum-free medium with mycozap in pre-coated T75 flasks (Greiner Bio-One, The Netherlands) as mentioned above and previously described (van Wetering et al., 2007), including some minor adaptations. Subsequently, PBEC were seeded at a density of 7000 cells/cm^2^ in passage 3-4 on 12- or 24-well plates (i.e. 1.9 cm^2^ or 3.8 cm^2^) (Corning Costar). The next day, PBEC were washed with HBSS (no calcium, no magnesium, no Phenol Red) (Gibco) and cultured in Lonza Bronchial Epithelial Basal Medium supplemented with Bronchial Epithelial Cell Growth Medium singlequots (except Gentamycin) (Lonza) and 1% penicillin/streptomycin (Gibco). Undifferentiated PBEC were cultured in submerged conditions (further referred to as S-PBEC) for ∼3 days upon 60-70% confluency. S-PBEC were starved for 4 h prior to treatment, by replacing the proliferation medium with starvation medium consisting of Lonza Bronchial Epithelial Basal Medium supplemented with Bronchial Epithelial Cell Growth Medium singlequots, except Gentamycin, Epidermal Growth Factor and Bovine Pituitary Extract (Lonza), and including 1% penicillin/streptomycin.

CSE was generated by using 3R4F research cigarettes (University of Kentucky, Lexington, KY, USA) according to the protocol previously described by Carp and Janoff (1978). In short, after removal of the filters, a cigarette was smoked until the filter paper line by bubbling air (2 ml/s) in HBSS (2 ml) (Gibco) using a linear pump following the regime of 5 s smoking 10 ml, 5 s pause. The ∼4 h-starved PBEC were exposed in technical triplicates to fresh sterile filtered CSE (1-2%) diluted in HBSS or control (HBSS; 0% CSE) in Lonza starvation medium for 4, 24 or 48 h.

### Submerged and ALI-PBEC culture and WCS treatment

After thawing PBEC of four non-COPD donors (based on pre-surgery spirometry; LUMC), cells were further expanded in supplemented keratinocyte serum-free medium with 1% penicillin/streptomycin, replacing primocin in T75 flasks as mentioned above and previously described (van Wetering et al., 2007). Next, 40,000 cells at passage 2 were transferred to 12-insert transwells coated with the same supplements as mentioned above. Apical and basal sides of transwells were filled with a mixture of 50% Bronchial Epithelial Cell Medium-basal (ScienCell, Sanbio) and 50% Dulbecco's modified Eagle medium (STEMCELL Technologies, Germany) (referred to as B/D medium), supplemented with 12.5 mM HEPES, bronchial epithelial cell growth supplement, 100 U/ml penicillin, 100 μg/ml streptomycin (all from ScienCell) and 2 mM glutaMAX (Thermo Fisher Scientific).

Undifferentiated PBEC were cultured in submerged conditions using medium supplemented with 1 nM of the synthetic retinoid EC23 (Tocris, Abingdon, UK) (referred to as S-PBEC) for ∼6 days to reach confluence before WCS or air exposure, or cultured at the ALI to induce mucociliary differentiation (referred to as ALI-PBEC) as previously described (Wang et al., 2020). After confluence was reached, the apical medium was removed, and cells were cultured at the ALI in B/D medium as described above with 50 nM EC23 for 2 weeks; three times a week the basal medium was refreshed and the apical side was washed with PBS to remove excess mucus.

For WCS exposure, S-PBEC or ALI-PBEC cultures on transwells were exposed to either fresh air (control) or WCS from one 3R4F research cigarette using an exposure chamber specifically designed for cell culture experiments as previously described (Amatngalim et al., 2018). In S-PBEC, apical medium was removed shortly before WCS exposure. Fresh air or WCS derived from one cigarette in the holder was pumped into the exposure chamber until one cigarette burned out. After that, fresh air was used to remove residual WCS from the chamber for 10 min. The weight difference of a filter placed between the pump and exposure chamber before and after exposure was recorded to measure the amount of smoke infused inside the exposure chamber. Approximately 2 mg CS-derived particles were deposited on the filter as determined by measuring the filter of different exposures. After WCS or air exposure, apical medium was added to undifferentiated cultures. Cultures were harvested at 6 h and 24 h after exposure according to experimental requirements.

For chronic WCS exposure and cessation, after reaching confluence on the inserts, ALI-PBEC were apically washed every day to remove mucus at 4 h prior to daily WCS exposure and exposed to either fresh air or WCS during differentiation at the ALI for a total of 14 days (chronic exposure model), followed by a cessation period of 10 days. Cells and basal medium were harvested on Day 14, 16, 19 and 23 to isolate proteins and mRNA as well as measure the levels of L-lactate.

### Separation of luminal and basal cell-enriched fractions

ALI-PBEC were separated into luminal and basal cell fractions at 6 h after WCS or air exposure as described previously, using calcium depletion followed by trypsinization (Amatngalim et al., 2018). Successful separation was identified by measuring gene expression of a basal cell marker (*TP63*) and an early progenitor cell marker (*KRT8*).

### RNA isolation, cDNA synthesis and real-time quantitative PCR analysis

CSE-treated undifferentiated PBEC were lysed after 4, 24 or 48 h in 200-400 µl RLT lysis buffer including 1% 2-Mercaptoethanol (Sigma-Aldrich) and processed according to the RNeasy^®^ Mini Kit manufacturer's protocol (74104 and 74106, Qiagen, USA). WCS-exposed S-PBEC or ALI-PBEC were lysed using RNA lysis buffer, and total RNA was robotically isolated using Maxwell^®^ 16 simply RNA tissue kit (Promega, The Netherlands). A NanoDrop ND 1000 UV-visible spectrophotometer (Isogen Life Sciences, The Netherlands or NanoDrop Technologies, USA) was used to analyze the quantity and purity of the RNA samples. Total RNA (CSE experiments, 25-140 ng; WCS experiments, 500 ng) was reverse transcribed using iScript™ cDNA synthesis method (Bio-Rad, The Netherlands). The cDNA was diluted in Milli-Q (CSE, 1:17.86-1:100; WCS, 1:50) in order to have an equal original input of 25 ng or 10 ng RNA per experiment and stored at −20°C until further analysis.

Expression of genes of interest was analyzed in all samples by real-time quantitative PCR (qPCR) by mixing diluted cDNA, target- and human-specific primers (Eurofins, The Netherlands or Invitrogen, USA) and a 2xSensiMix™ SYBR^®^ & Fluorescein Kit (Bioline, The Netherlands) or IQ SYBR Green Supermix (Bio-Rad) in white 384-multiwell plates (Roche, Switzerland or BIOplastics BV, The Netherlands). Subsequently, the thermal cycling protocol (10 min at 95°C, 55 cycles of 10 s at 95°C, 20 s at 60°C) was run on a LightCycler 480 machine (Roche). The following software programs were used to perform melt curve and gene expression analysis: LightCycler480 software (Roche) and LinRegPCR software 2014.x (The Netherlands), respectively. Moreover normalization of the expression of mRNA transcripts of interest was conducted using GeNorm software 3.4 (Primerdesign, USA), which calculated a correction factor based on the expression of a combination of reference genes [*ACTB*, *B2M*, *PPIA*, *RPL13A*, *ATP5B* (also known as *ATP5F1B*)]. Used target and human-specific primer sequences are listed in Table S1.

### DNA isolation and mtDNA copy number analysis

Following exposures, CSE was kept with the cells for 24 h or 48 h, or cells were cultured for 6 h or 24 h after short WCS treatment. Next, PBEC were lysed in 250-400 µl lysis buffer [0.1 M Tris-HCI pH 8.5, 0.005 M EDTA pH 8.0, 0.2% (w/v) sodium dodecyl sulphate, 0.2 M NaCl] at room temperature. To isolate DNA, addition of Proteinase K (10 mg/ml) (Qiagen) to the lysates (1:50) was required, followed by overnight incubation at 55°C. The next day, lysates were centrifuged at 20,000 ***g*** for 15 min. Thereafter, 500 µl isopropanol was added to the supernatant, facilitating DNA precipitation by vigorously shaking. Following two washes of the DNA pellets with 70% ethanol, dry DNA pellets were dissolved in 125 µl TE buffer (10 mM Tris-HCI pH 8.0, 1 mM EDTA). The DNA samples were subsequently incubated at 55°C for 2 h, overnight at 4°C and finally stored at −20°C until use. DNA quantity and purity was determined using the NanoDrop ND 1000 UV-visible spectrophotometer. DNA samples were diluted in TE buffer (1:50) followed by qPCR analysis (see ‘RNA isolation, cDNA synthesis and real-time quantitative PCR analysis’ section). mtDNA copy numbers were assessed by investigating the ratio of the expression of mtDNA, mitochondrially encoded cytochrome C oxidase II (*MT-CO2*) and genomic DNA, *ACTB* (Table S1).

### Western blotting

Whole-cell lysates for western blotting were generated by lysis of treated PBEC in 200 µl whole-cell lysis buffer (20 mM Tris-HCl pH 7.4, 150 mM NaCl, 1% Nonidet P40 in Milli-Q) or Pierce RIPA buffer (Thermo Fisher Scientific), including fresh PhosSTOP Phosphatase and cOmplete, Mini, EDTA-free protease inhibitor cocktail tablets (both Roche). The whole-cell lysates were rotated and subsequently centrifuged at 20,000 ***g*** for 30 min at 4°C. Assessment of the total protein content in the whole-cell lysate fraction was conducted according to the manufacturer's protocol of the Pierce™ BCA Protein Assay Kit range 20-2000 µg/ml (Thermo Fisher Scientific). The whole-cell lysate supernatant was consecutively diluted to similar concentration within experiments (range 0.0667-1 µg/µl) in a final concentration of 1× Laemmli buffer [0.25 M Tris-HCl pH 6.8, 8% (w/v) sodium dodecyl sulphate, 40% (v/v) glycerol, 0.4 M dithiothreitol, 0.02% (w/v) Bromophenol Blue], boiled at 100°C for 5 min and stored at −80°C pending analysis.

Samples (1-10 µg of protein per lane) and at least two protein ladders (Precision Plus Protein™ All Blue Standards #161-0373, Bio-Rad) were run on each gel in 1× MES running buffer (Bio-Rad) on a Criterion XT Precast 4-12 or 12% Bis-Tris gel (Bio-Rad). Separation of the proteins was achieved by electrophoresis (100-130 V for 1 h), followed by electroblotting (Bio-Rad Criterion Blotter) (100 V for 1 h) to transfer the proteins from the gel to a 0.45 µM nitrocellulose transfer membrane (Bio-Rad). To quantify total protein content, the nitrocellulose membranes were stained using 0.2% (w/v) Ponceau S in 1% (v/v) acetic acid (Sigma-Aldrich) for 5 min, followed by a Milli-Q wash and imaging using the Amersham™ Imager 600 (GE Healthcare, The Netherlands). After removal of the Ponceau S staining, non-specific binding sites on the membranes were blocked for 1 h in 3% (w/v) non-fat dry milk (Campina, The Netherlands) in Tween 20 Tris-buffered saline [TBST; 20 mM Tris, 137 mM NaCl, 0.1% (v/v) Tween 20, pH 7.6]. Subsequently, a TBST wash was followed by overnight incubation of the membranes at 4°C with a target-specific primary antibody (Table S2) diluted 1:500-1:2000 in TBST with 3% (w/v) BSA or non-fat dry milk. The next day, membranes were washed and incubated with a horseradish peroxidase-conjugated secondary antibody (Table S2) diluted 1:10,000 in 3% (w/v) non-fat dry milk in TBST for 1 h at room temperature. Thereafter, membranes were washed again and incubated for 3 min with either 0.25× Supersignal West FEMTO or 0.5× Supersignal West PICO Chemiluminescent Substrate (Thermo Fisher Scientific) to visualize the target proteins using the Amersham™ Imager 600. Quantification of images was performed using Image Quant software (GE Healthcare). Absolute protein quantification was calculated by correcting for total protein loading content assessed by Ponceau S staining over the entire size range of proteins (10-250 kDa). For western blot analysis, in each of the models described in Fig. 1, based on the respective molecular mass, the following proteins were loaded on the same gel, implying that, for the quantification of these proteins, normalization was based on the same Ponceau S staining (Gel I: HK2, DNM1L, OXPHOS, SQSTM1; Gel II: PRKN, BNIP3L, BNIP3, FUNDC1, PINK1, MAP1LC3B; Gel III: PPARGC1A, NRF1, TFAM, GABARAPL1, ESRRA). The western blot images presented in the figures of this paper have been equally adjusted for brightness and contrast throughout the picture. Selected images reflecting changes in one PBEC donor are representative for the group of donors/experiment (*n*=3/4 donors/experiment).

### Metabolic enzyme activity assays

Metabolic enzyme activity lysates were generated by lysis of treated PBEC in 100 µl 0.5% Triton X-100 (Sigma-Aldrich) for 15 min on ice, followed by scraping (on ice). Subsequently, the lysates were centrifuged at 20,000 ***g*** for 2 min at 4°C. Supernatants were aliquoted for protein analysis or diluted in 5% BSA in Milli-Q (1:4) for metabolic enzyme activity analysis, both stored at −80°C. Total protein content in the supernatant fraction was evaluated following the manufacturer's protocol of the Pierce™ BCA Protein Assay Kit range 20-2000 µg/ml.

After running the three assays [citrate synthase, HADH and PFK1], analysis of the samples was spectrophotometrically conducted at 340 nM (HADH/PFK1) or 412 nM (citrate synthase) at 37°C using a Multiskan Spectrum plate reader (Thermo Labsystems, The Netherlands). Enzyme activity was calculated by slope determination and correction for total protein content of the samples. Details of the analyses were as follows:

#### Citrate synthase

Citrate synthase activity was assessed as previously described (IUBMB Enzyme Nomenclature EC 2.3.3.1) (Shepherd and Garland, 1969) by mixing undiluted samples with reagent (100 mM Tris, 0.1 mM DNTB, 40 µM acetyl coenzyme A), followed by initiation of the reaction by addition of oxaloacetic acid (25 mM).

#### HADH

HADH enzyme activity was assessed as previously described (IUBMB Enzyme Nomenclature EC 1.1.1.35) (Bergmeyer et al., 1974). After mixing the undiluted samples with reagent (0.22 mM NADH, 100 mM tetrapotassium pyrophosphate pH 7.3), the reaction was initiated by addition of 2.3 mM acetoacetyl-CoA.

#### PFK1

As previously described (IUBMB Enzyme Nomenclature EC 2.7.1.11) (Ling et al., 1966), PFK1 enzyme activity was evaluated by mixing undiluted samples with reagent (48.8 mM Tris, 7.4 mM MgCl_2_.6H_2_O, 74 mM KCl, 384 μM KCN, 2.8 mM ATP, 1.5 mM DTT, 0.3 mM NADH, 0.375 U/ml aldolase, 0.5625 U/ml glycerol-3-phosphate dehydrogenase and 7.425 U/ml triose phosphate isomerase, pH 8.0). To initiate the reaction, fructose-6-phosphate (30.6 mM) in Tris buffer (49.5 mM, pH 8.0) was added to the mix of undiluted sample and reagent.

### Lactate assay

Levels of L-lactate in apical and basal medium collected from S-PBEC and the basal medium of ALI-PBEC at 24 h after acute/chronic WCS exposure were measured using a Lactate Colorimetric/Fluorometric Assay Kit (K607-100, BioVision, USA). In brief, 50 µl diluted samples in Lactate Assay Buffer were mixed with 50 µl Reaction Mix (Lactate Enzyme Mix, Probe and Lactate Assay Buffer) and incubated for 30 min. The absorbance at OD 570 nm was measured in a microplate reader (Bio-Rad).

### Immunofluorescence staining and confocal microscopy

Immunofluorescence staining of ALI-PBEC cultures on inserts was performed as mentioned before (Wang et al., 2020). At indicated time points after acute WCS exposure, membranes with attached ALI-PBEC were washed and PBEC fixed in 4% (w/v) paraformaldehyde in PBS for 30 min at room temperature. Membranes were washed once and stored in PBS at 4°C until use. Before staining for intracellular antigens, ice-cold methanol was added for 10 min at 4°C. Blocking and permeabilization buffer for non-specific binding sites was PBS/1% (w/v) BSA/0.3% (w/v) Triton-X-100 (PBT) buffer. After incubation for 30 min at 4°C, specific binding sites were stained with rabbit anti-LC3B antibody (1:100; Cell Signaling Technology, USA) together with mouse anti-MUC5AC antibody (1:200; Thermo Fisher Scientific), anti-acetylated α-tubulin antibody (1:500; Sigma-Aldrich), anti-CC-10 antibody (1:50; Hycult Biotech, The Netherlands) or anti-NGFR antibody (1:100; Abcam, UK) for 1 h at room temperature. After washing, secondary antibodies (donkey anti-rabbit and donkey anti-mouse Alexa Flour antibodies; all diluted 1:200, Thermo Fisher Scientific) and 4′,6-diamidino-2-phenylindole (DAPI; 1:50, Sigma-Aldrich) were added to the cells in the dark for 30 min at room temperature. Next, membranes with ProLong™ Gold Antifade Mountant (Thermo Fisher Scientific) were placed on glass slides and covered with a coverslip. Slides were viewed using a TCS SP8 confocal microscope (Leica Microsystems, Germany) at 630× original magnification.

### Statistical analysis

Prism 8.0.1 software (GraphPad, USA) was used to perform statistical analyses and graph the data. Data are presented as mean fold change of independent donors compared to control (air, 0% CSE or Day 14)±s.e.m. In the CSE-exposed S-PBEC experiments, each donor reflects the mean of technical triplicates. Outliers were excluded based on quality assessment of gene expression melt curve/peak analysis using LightCycler480 Software (Roche) and western blot analysis. Statistical testing of differences between acute WCS and air exposures in S-PBEC or ALI-PBEC was performed using a two-tailed paired parametric *t*-test. Moreover, a two-tailed paired parametric *t*-test was conducted to test differences in WCS versus air exposures after smoking cessation in ALI-PBEC on each day (e.g. WCS Day 14 versus air control). If comparison of various groups was required in case of the CSE exposure (CSE 1% or 2% versus 0% CSE) or in WCS chronic smoking cessation experiments (WCS Day 16, 19, 23 versus WCS Day 14), assuming a Gaussian distribution and using Geisser–Greenhouse correction, one-way ANOVA (matched/repeated measures) followed by Sidak's post-hoc test for multiple comparisons was conducted, and, in the case of missing values, mixed-effects modeling was performed. Statistical significance was considered if *P*<0.05 and a trend was indicated if *P*<0.1.

## Supplementary Material

Supplementary information
