# Supplementary material for: Dysregulated mitochondrial metabolism upon cigarette smoke exposure in various human bronchial epithelial cell models
Source: Dis Model Mech. 2022 Mar 28;15(3):dmm049247. doi: 10.1242/dmm.049247 (PMC8990921; doi:10.1242/dmm.049247)
Supplement: Supplementary information [file dmm-15-049247-s1.pdf]

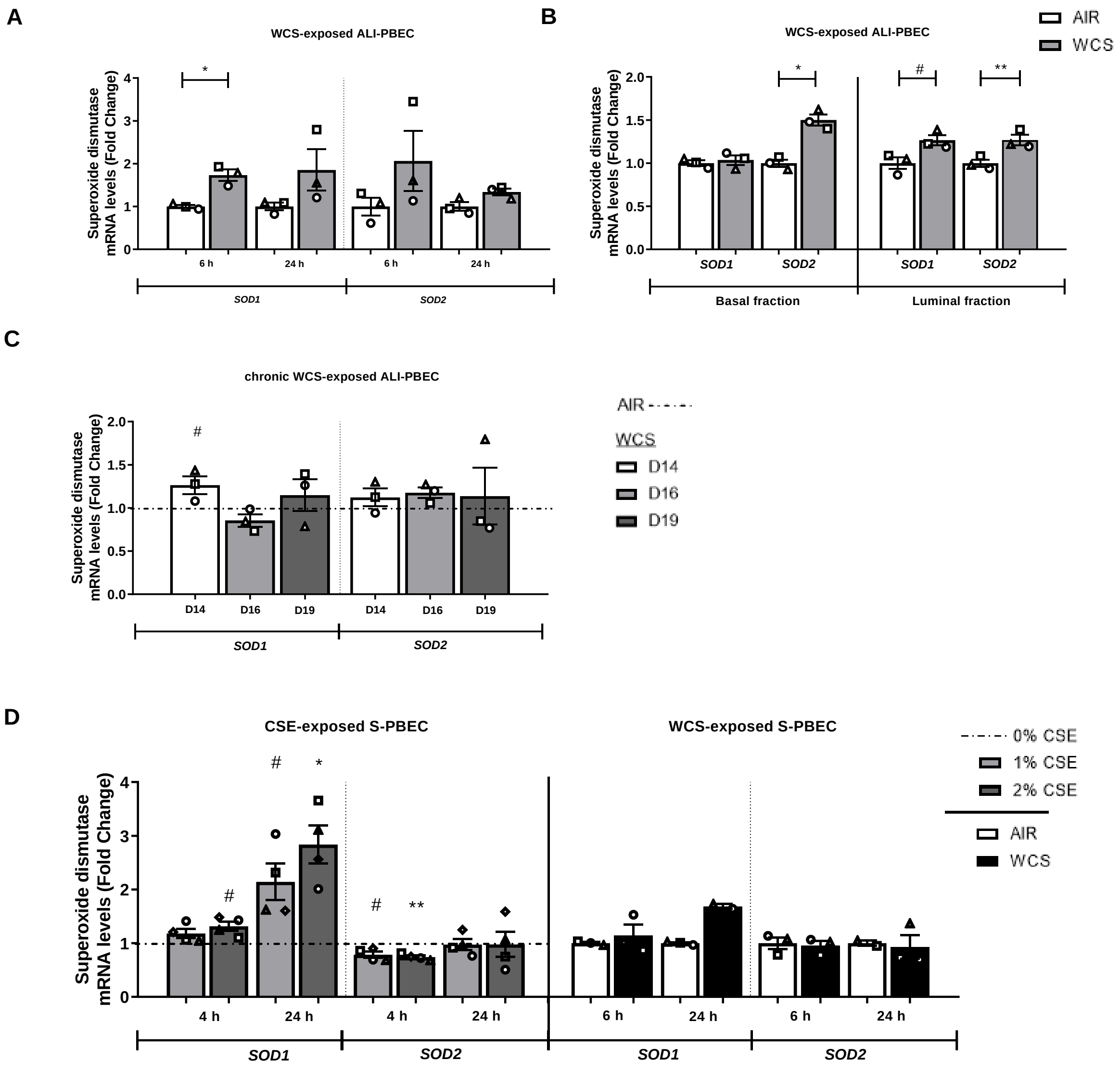

Fig. S1. Increase in expression of genes involved in anti-oxidant response after CS exposure in both ALI-and S-PBEC. After 2-weeks of differentiation, ALI-PBEC were exposed to fresh air or WCS from one 3R4F cigarette (University of Kentucky, 2 mg) and whole-cell lysates were harvested after 6h and 24 h, and the basal and luminal fractions were harvested only at 6 h post-exposure (n=3 donors/group). Gene expression of *SOD1* and *SOD2* in whole-cell lysates of ALI-PBEC (**A**) and separated fractions (**B**) were analyzed by real-time qPCR. ALI-PBEC were 1x daily exposed to fresh air or WCS from one 3R4F cigarette (University of Kentucky, 2 mg) during differentiation for 14 days followed by a cessation period up to 10 days. Cells were harvested on Day 14 (24 hours after the last WCS exposure), 16 and 19 (n=3 donors/group). mRNA levels of *SOD1* and *SOD2* **(C)** were analyzed in whole-cell lysates. Undifferentiated S-PBEC were treated with CSE from one 3R4F cigarette (University of Kentucky) diluted in HBSS (0-1-2%) in Lonza starvation medium for 4 h or 24 h (n=4 donors/group) or undifferentiated S-PBEC cultured on transwells were exposed, after removal of apical medium, to fresh air or WCS from one 3R4F cigarette (University of Kentucky, 2 mg) followed by harvesting of whole-cell lysates

after 6 h or 24 h recovery (n=2-3 donors/group). mRNA levels of *SOD1* and *SOD2* **(D)** were analyzed in whole-cell lysates. Data are presented as mean fold change compared to control (air, 0% CSE or WCS Day 14) ± s.e.m.. Independent donors are represented by open circles, triangles, squares or diamonds. In case of the CSE-exposed S-PBEC experiments, the symbols reflect the mean of technical triplicates. Statistical differences between WCS versus air or WCS versus air after smoking cessation in ALI-PBEC on each day (e.g., WCS Day 14 versus air). were tested using a two-tailed paired parametric *t*-test. If comparison of various groups was required in case of the CSE exposure (CSE 1% or 2% versus 0% CSE) or in WCS chronic smoking cessation experiments (WCS Day 16, 19 versus WCS Day 14), an one-way ANOVA (matched/repeated measures) followed by Sidak's post-hoc test for multiple comparisons was conducted, and in case of missing values the mixed-effects models was performed. Statistical significance is indicated as $^{\#}p<0.1$, $^{*}p<0.05$ and $^{**}p<0.01$ compared to control (air, 0% CSE or WCS Day 14).

Disease Models & Mechanisms • Supplementary information

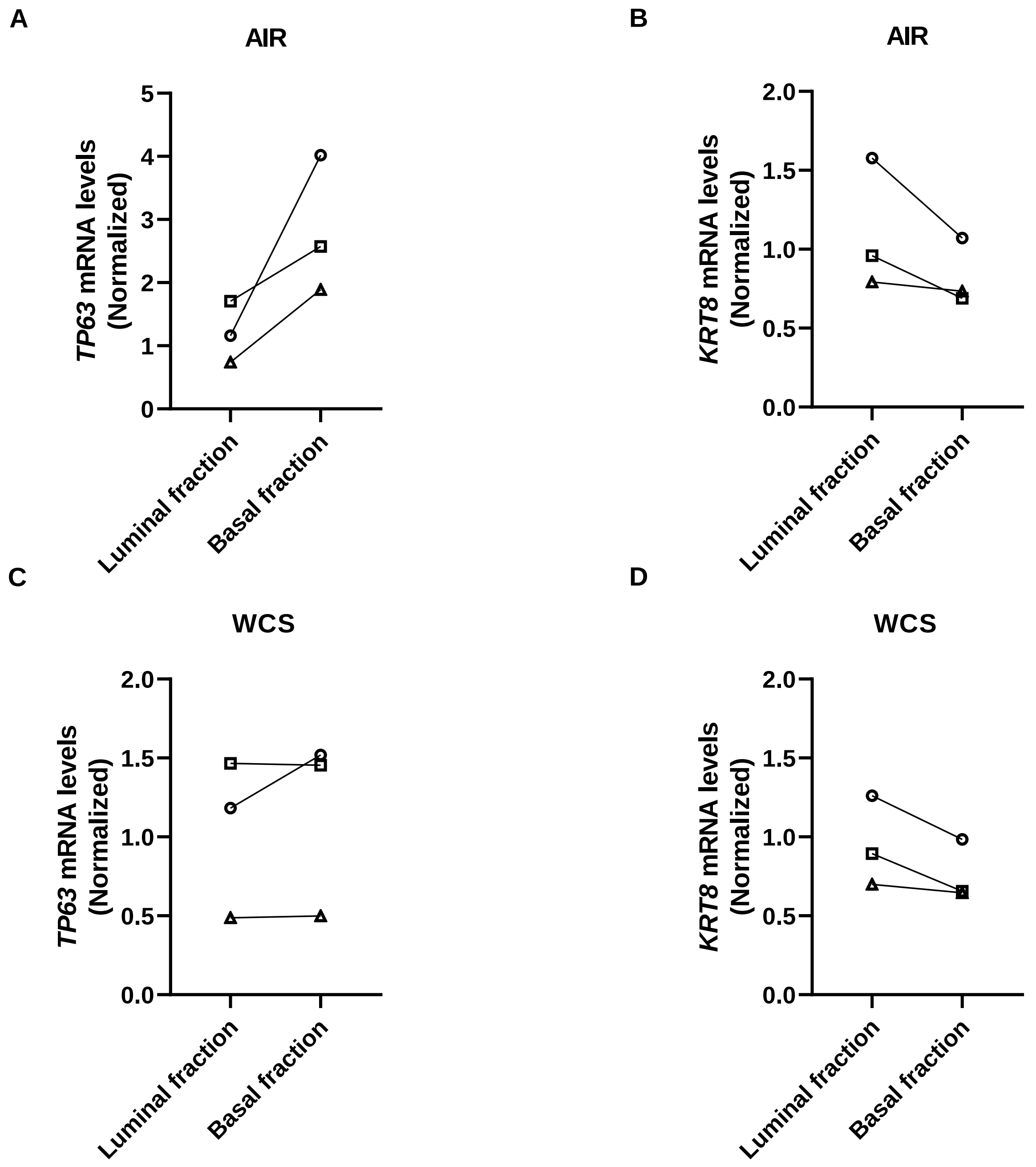

Disease Models & Mechanisms • Supplementary information

Fig. S2. Validation of separation of basal and luminal cell fractions from ALI-PBEC. After 2-weeks of differentiation, ALI-PBEC were exposed to fresh air or WCS from one 3R4F cigarette (University of Kentucky, 2 mg) and separated into luminal and basal cell fractions at 6 h post-exposure using calcium depletion followed by trypsinization (n=3 donors/group). Independent donors are represented by open circles, triangles or squares. Successful separation was identified by measuring gene expression of basal cells marker (Tp63) **(A, C)** and early progenitor cell marker (cytokeratin-8, KRT8) **(B, D)**.

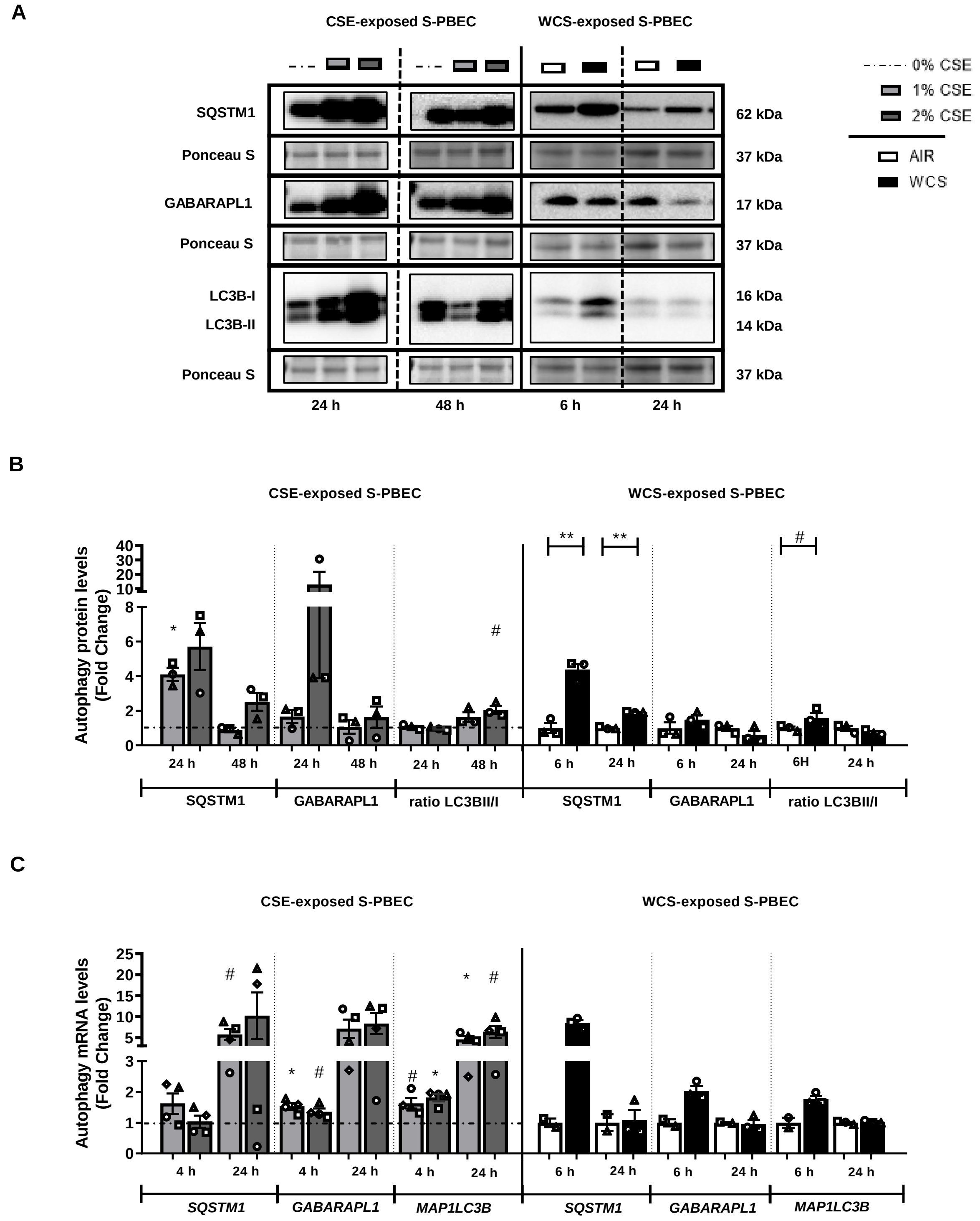

Disease Models & Mechanisms • Supplementary information

Fig. S3. Increase in abundance of key constituents involved in autophagy following acute CSE or WCS exposure in S-PBEC. Undifferentiated S-PBEC were treated with CSE from one 3R4F cigarette (University of Kentucky) diluted in HBSS (0-1-2%) in Lonza starvation medium for 4 h, 24 h or 48 h (n=3-4 donors/group) or undifferentiated S-PBEC cultured on transwells were exposed, after removal of apical medium, to fresh air or WCS from one 3R4F cigarette (University of Kentucky, 2 mg) followed by harvesting of whole-cell lysates after 6 h or 24 h recovery (n=2-3 donors/group). Protein **(A, B)** as well as transcript abundance **(C)** of autophagy regulators SQSTM1, GABARAPL1 and ratio LC3BII/I or MAP1LC3B were measured by western blot and real-time qPCR. Representative western blots, including representative parts of the Ponceau S staining, are shown. Data are presented as mean fold change compared to control (0% CSE or air) ± s.e.m.. Independent donors are represented by open circles, triangles, squares or diamonds. In case of the CSE-exposed S-PBEC experiments, the symbols reflect the mean of technical triplicates. Statistical differences between the various CSE exposure groups (CSE 1% or 2% versus 0% CSE) were tested using an one-way ANOVA (matched/repeated measures) followed by Sidak's post-hoc test for multiple comparisons, and in case of missing values the mixed-effects models was performed. WCS versus air was tested using a two-tailed paired parametric *t*-test. Statistical significance is indicated as $^{\#}$p<0.1, *p<0.05 and **p<0.01 compared to control (0% CSE or air).

Disease Models & Mechanisms • Supplementary information

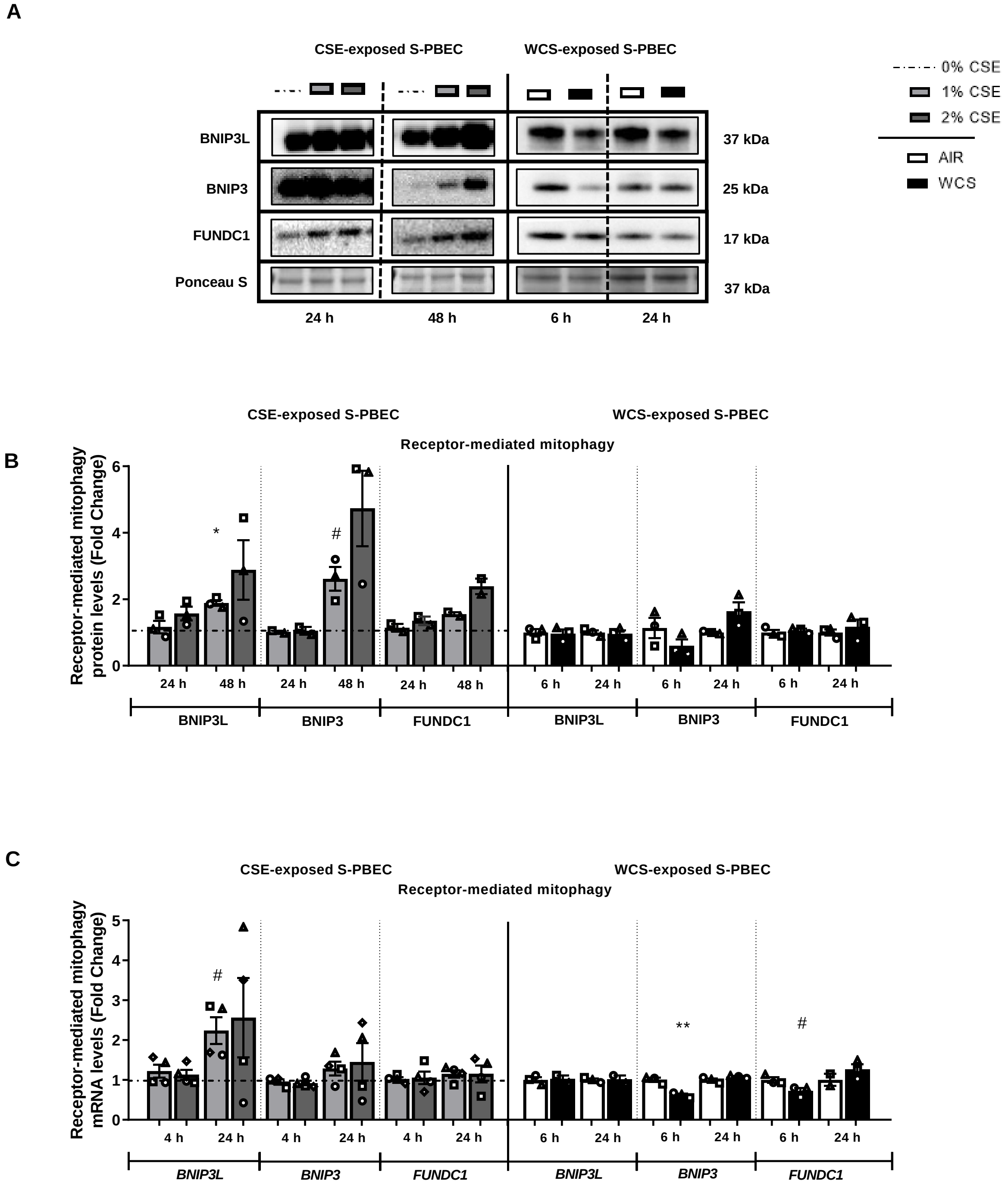

Disease Models & Mechanisms • Supplementary information

Fig. S4. Upregulation of constituents of the receptor-mediated mitophagy machinery in CS-exposed S-PBEC. Undifferentiated S-PBEC were treated with CSE from one 3R4F cigarette (University of Kentucky) diluted in HBSS (0-1-2%) in Lonza starvation medium for 4 h, 24 h or 48 h (n=2-4 donors/group) or undifferentiated S-PBEC cultured on transwells were exposed, after removal of apical medium, to fresh air or WCS from one 3R4F cigarette (University of Kentucky, 2 mg) followed by harvesting of whole-cell lysates after 6 h or 24 h recovery (n=2-3 donors/group). Protein **(A, B)** and mRNA levels **(C)** of regulators involved in receptor-mediated mitophagy (BNIP3L, BNIP3, FUNDC1) were analyzed in whole-cell lysates. Representative western blots, including representative parts of the Ponceau S staining, are shown. Data are presented as mean fold change compared to control (0% CSE or air) ± s.e.m.. Independent donors are represented by open circles, triangles, squares or diamonds. In case of the CSE-exposed S-PBEC experiments, the symbols reflect the mean of technical triplicates. Statistical differences between the various CSE exposure groups (CSE 1% or 2% versus 0% CSE) were tested using an one-way ANOVA (matched/repeated measures) followed by Sidak's post-hoc test for multiple comparisons, and in case of missing values the mixed-effects models was performed. WCS versus air was tested using a two-tailed paired parametric *t*-test. Statistical significance is indicated as $^{\#}$p<0.1, *p<0.05 and **p<0.01 compared to control (0% CSE or air).

Disease Models & Mechanisms • Supplementary information

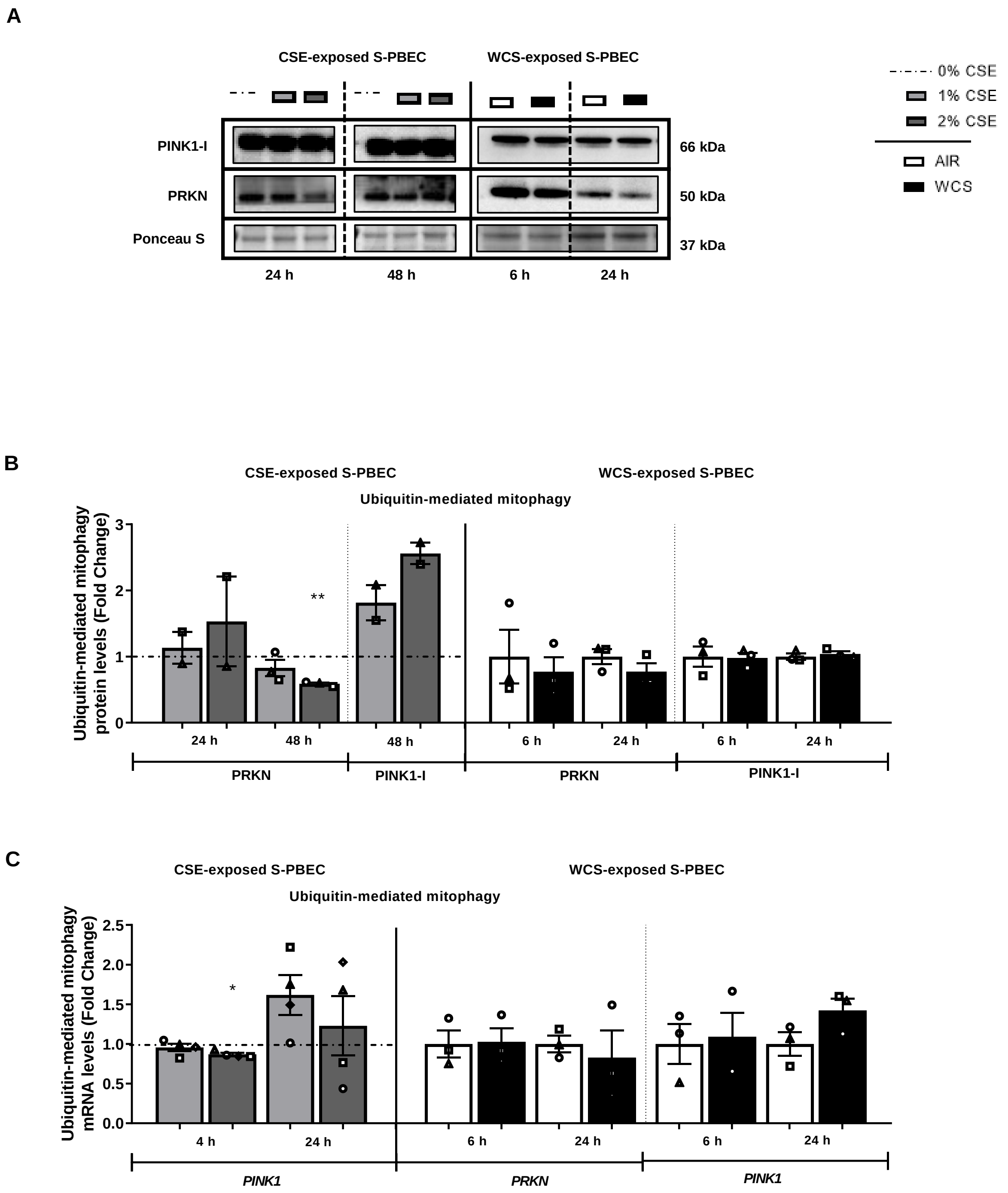

Disease Models & Mechanisms • Supplementary information

Fig. S5. Modulation of ubiquitin-mediated mitophagy markers in CS-exposed S-PBEC. Undifferentiated S-PBEC were treated with CSE from one 3R4F cigarette (University of Kentucky) diluted in HBSS (0-1-2%) in Lonza starvation medium for 4 h, 24 h or 48 h (n=2-4 donors/group) or undifferentiated S-PBEC cultured on transwells were exposed, after removal of apical medium, to fresh air or WCS from one 3R4F cigarette (University of Kentucky, 2 mg) followed by harvesting of whole-cell lysates after 6 h or 24 h recovery (n=3 donors/group). Protein **(A, B)** and mRNA levels **(C)** of regulators involved in ubiquitin-mediated mitophagy (PRKN, PINK1) were analyzed in whole-cell lysates. Western blot analysis revealed one distinct band for PINK1 protein corresponding with expected molecular weight for PINK1-I (66 kDa). Representative western blots, including representative parts of the Ponceau S staining, are shown. Data are presented as mean fold change compared to control (0% CSE or air) ± s.e.m.. Independent donors are represented by open circles, triangles, squares or diamonds. In case of the CSE-exposed S-PBEC experiments, the symbols reflect the mean of technical triplicates. Statistical differences between the various CSE exposure groups (CSE 1% or 2% versus 0% CSE) were tested using an one-way ANOVA (matched/repeated measures) followed by Sidak's post-hoc test for multiple comparisons, and in case of missing values the mixed-effects models was performed. WCS versus air was tested using a two-tailed paired parametric *t*-test. Statistical significance is indicated as *p<0.05 and **p<0.01 compared to control (0% CSE or air).

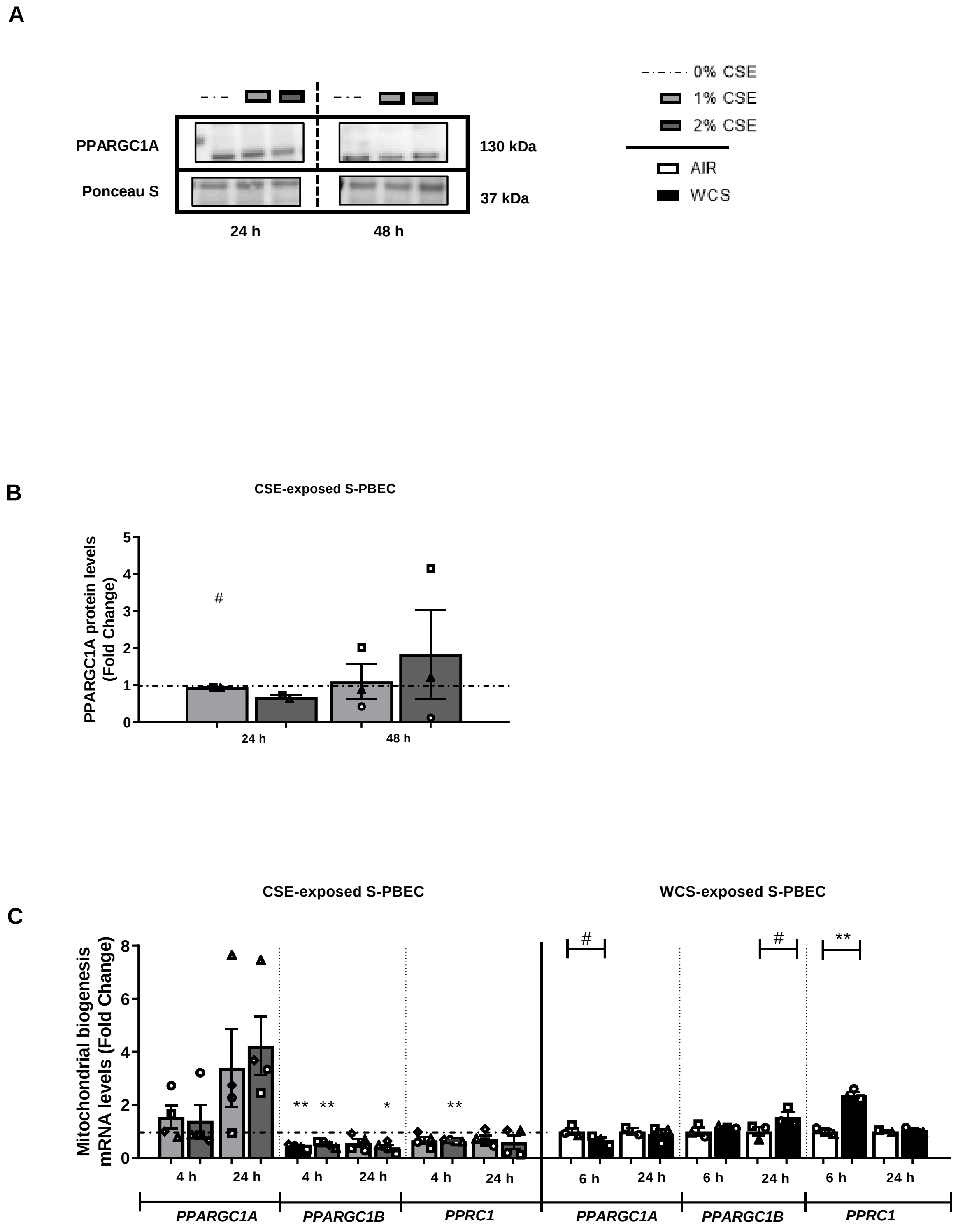
A
PPARGC1A
Ponceau S
130 kDa
37 kDa
24 h
48 h
0% CSE
1% CSE
2% CSE
AIR
WCS
B
CSE-exposed S-PBEC
PPARGC1A protein levels (Fold Change)
#
24 h
48 h
C
CSE-exposed S-PBEC
WCS-exposed S-PBEC
Mitochondrial biogenesis mRNA levels (Fold Change)
4 h
24 h
6 h
PPARGC1A
PPARGC1B
PPRC1

Fig. S6. Alterations in expression of transcript levels of transcriptional co-activators of the PPARGC1 network in response to acute CS exposure in S-PBEC. Undifferentiated S-PBEC were treated with CSE from one 3R4F cigarette (University of Kentucky) diluted in HBSS (0-1-2%) in Lonza starvation medium for 4 h, 24 h or 48 h (n=2-4 donors/group) or undifferentiated S-PBEC cultured on transwells were exposed, after removal of apical medium, to fresh air or WCS from one 3R4F cigarette (University of Kentucky, 2 mg) followed by harvesting of whole-cell lysates after 6 h or 24 h recovery (n=2-3 donors/group). Protein **(A, B)** as well as transcript abundance **(C)** of transcriptional co-activators involved in the PPARGC1 network (PPARGC1A, PPARGC1B, PPRC1) are presented. Representative western blots, including representative parts of the Ponceau S staining, are shown. Data are presented as mean fold change compared to control (0% CSE or air) ± s.e.m.. Independent donors are represented by open circles, triangles, squares or diamonds. In case of the CSE-exposed S-PBEC experiments, the symbols reflect the mean of technical triplicates. Statistical differences between the various CSE exposure groups (CSE 1% or 2% versus 0% CSE) were tested using an one-way ANOVA (matched/repeated measures) followed by Sidak's post-hoc test for multiple comparisons, and in case of missing values the mixed-effects models was performed. WCS versus air was tested using a two-tailed paired parametric *t*-test. Statistical significance is indicated as $^{\#}p<0.1$, $^{*}p<0.05$ and $^{**}p<0.01$ compared to control (0% CSE or air).

Disease Models & Mechanisms • Supplementary information

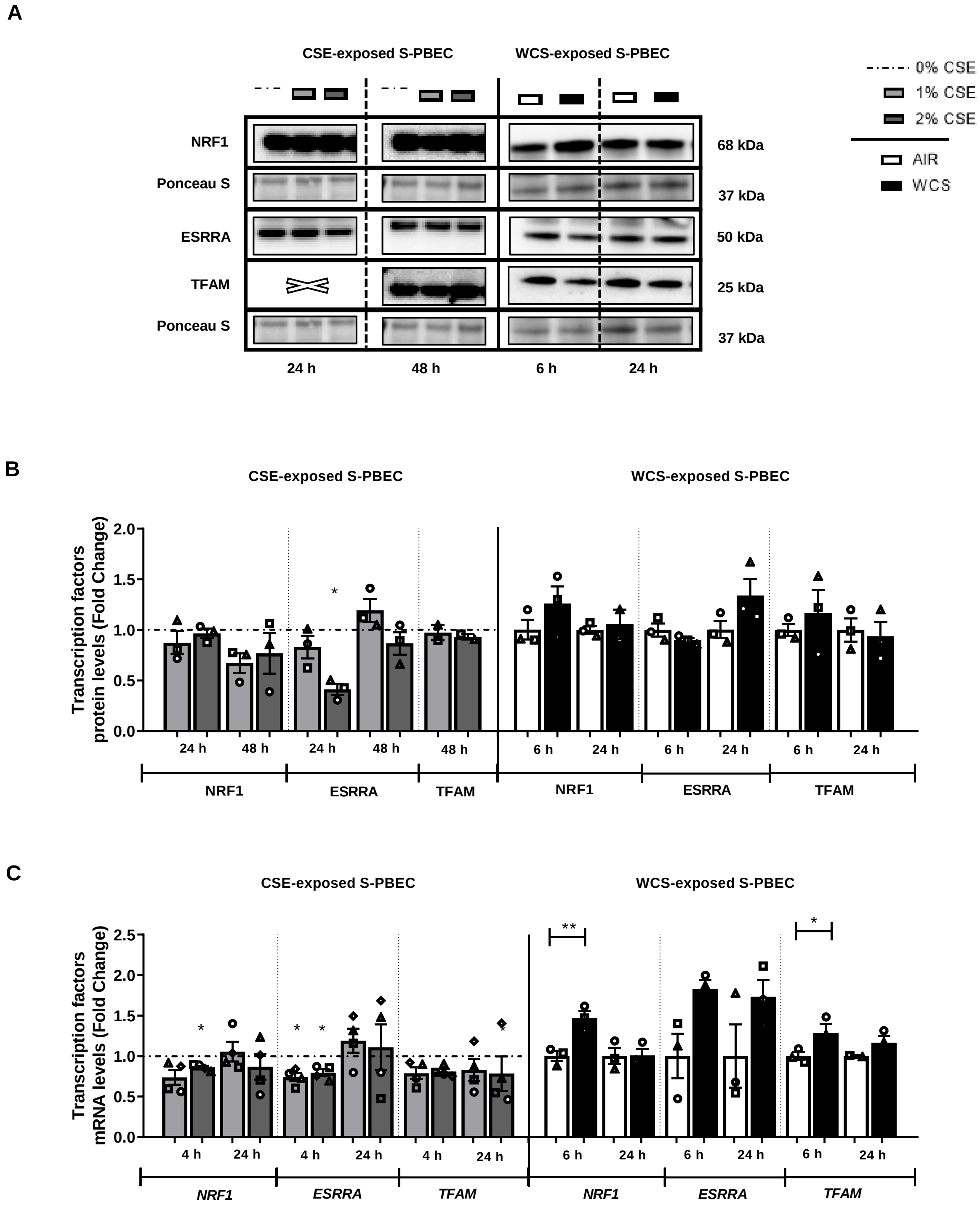

Disease Models & Mechanisms • Supplementary information

Fig. S7. Changes in the abundance of PPARGC1-coactivated transcription factors after CS exposure in S-PBEC. Undifferentiated S-PBEC were treated with CSE from one 3R4F cigarette (University of Kentucky) diluted in HBSS (0-1-2%) in Lonza starvation medium for 4 h, 24 h or 48 h (n=3-4 donors/group) or undifferentiated S-PBEC cultured on transwells were exposed, after removal of apical medium, to fresh air or WCS from one 3R4F cigarette (University of Kentucky, 2 mg) followed by harvesting of whole-cell lysates after 6 h or 24 h recovery (n=2-3 donors/group). Protein **(A, B)** and mRNA levels **(C)** of PPARGC1-coactivated transcription regulators, NRF1, ESRRA and TFAM, were measured by western blotting or real-time qPCR. Representative western blots, including representative parts of the Ponceau S staining, are shown. Data are presented as mean fold change compared to control (0% CSE or air) ± s.e.m.. Independent donors are represented by open circles, triangles, squares or diamonds. In case of the CSE-exposed S-PBEC experiments, the symbols reflect the mean of technical triplicates. Statistical differences between the various CSE exposure groups (CSE 1% or 2% versus 0% CSE) were tested using an one-way ANOVA (matched/repeated measures) followed by Sidak's post-hoc test for multiple comparisons, and in case of missing values the mixed-effects models was performed. WCS versus air was tested using a two-tailed paired parametric *t*-test. Statistical significance is indicated as *$p<0.05$ and **$p<0.01$ compared to control (0% CSE or air).

Disease Models & Mechanisms • Supplementary information

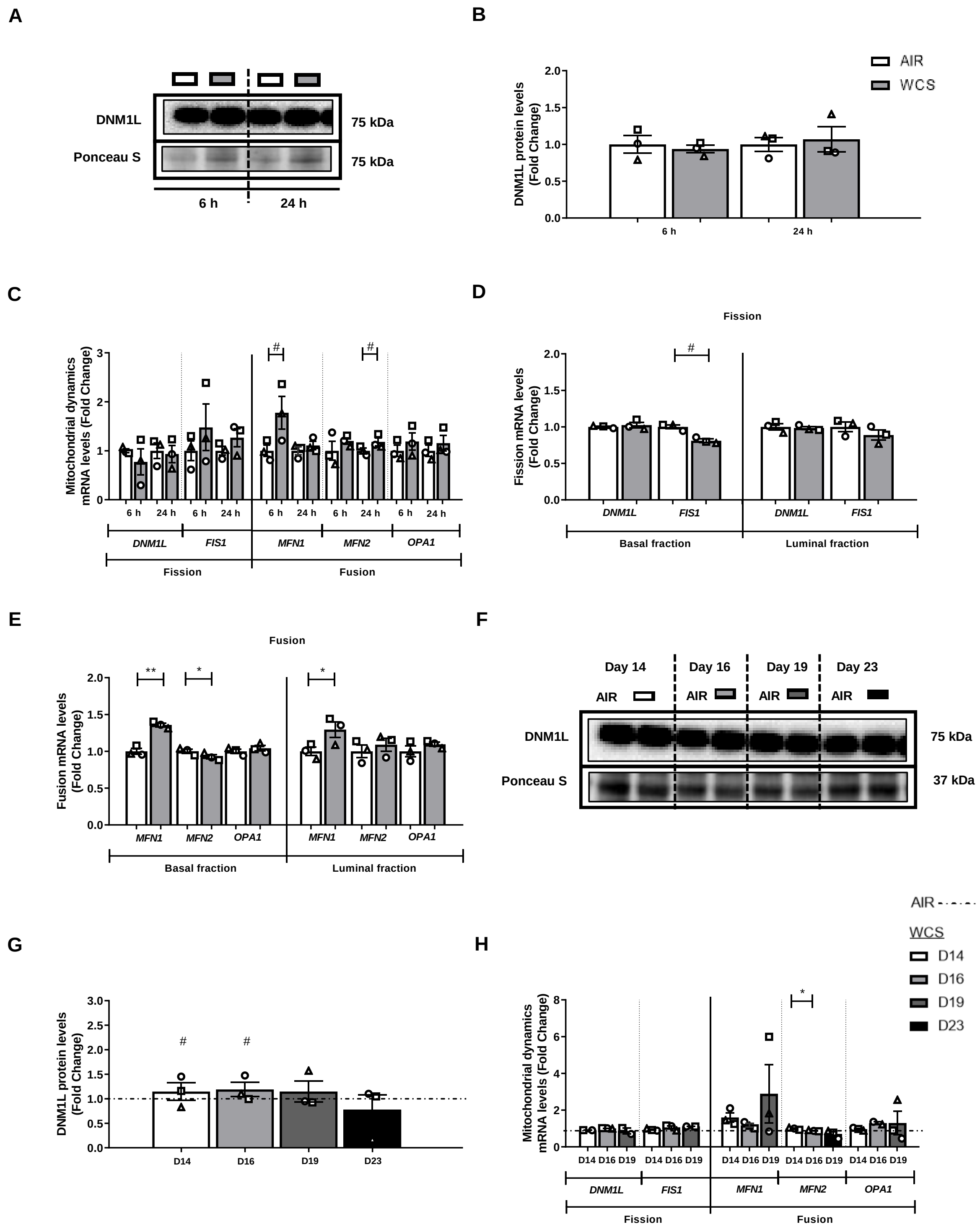

Disease Models & Mechanisms • Supplementary information

Fig. S8. Changes in mitochondrial dynamics markers in response to WCS exposure in ALI-PBEC. After 2-weeks of differentiation, ALI-PBEC were exposed to fresh air or WCS from one 3R4F cigarette (University of Kentucky, 2 mg) and whole-cell lysates were harvested after 6h and 24 h, and the basal and luminal fractions were harvested only at 6 h post-exposure (n=3 donors/group). Protein **(A, B)** and mRNA levels **(C, D, E)** of fission- and fusion-associated markers were analyzed in whole-cell lysates or basal/luminal cell fractions post-exposure. Data are presented as mean fold change compared to control (air) ± s.e.m.. Independent donors are represented by open circles, triangles or squares. Statistical differences between WCS versus air were tested using a two-tailed paired parametric *t*-test, $^{\#}p<0.1$, $^{*}p<0.05$ and $^{**}p<0.01$. ALI-PBEC were 1x daily exposed to fresh air or WCS from one 3R4F cigarette (University of Kentucky, 2 mg) during differentiation for 14 days followed by a cessation period up to 10 days. Cells were harvested on Day 14 (24 h after the last exposure), 16, 19 and 23 (n=2-3 donors/group). Abundance of DNM1L protein **(F, G)** and transcript abundance of fission and fusion regulators **(H)** are shown. Representative western blots, including representative parts of the Ponceau S staining, are shown. Data are presented as mean fold change compared to control (air or WCS Day 14) ± s.e.m.. Independent donors are represented by open circles, triangles or squares. Statistical differences between WCS versus air after smoking cessation in ALI-PBEC on each day was tested using a two-tailed paired parametric *t*-test (e.g., WCS Day 14 versus air). Comparison of various groups to test the difference of WCS Day 16, 19, 23 versus Day 14 in WCS chronic smoking cessation experiments was conducted using an one-way ANOVA followed by Sidak's post-hoc test for multiple comparisons, and in case of missing values the mixed-effects models was performed. Statistical significance is indicated as $^{\#}p<0.1$ and $^{*}p<0.05$ compared to control (air or WCS Day 14).

Disease Models & Mechanisms • Supplementary information

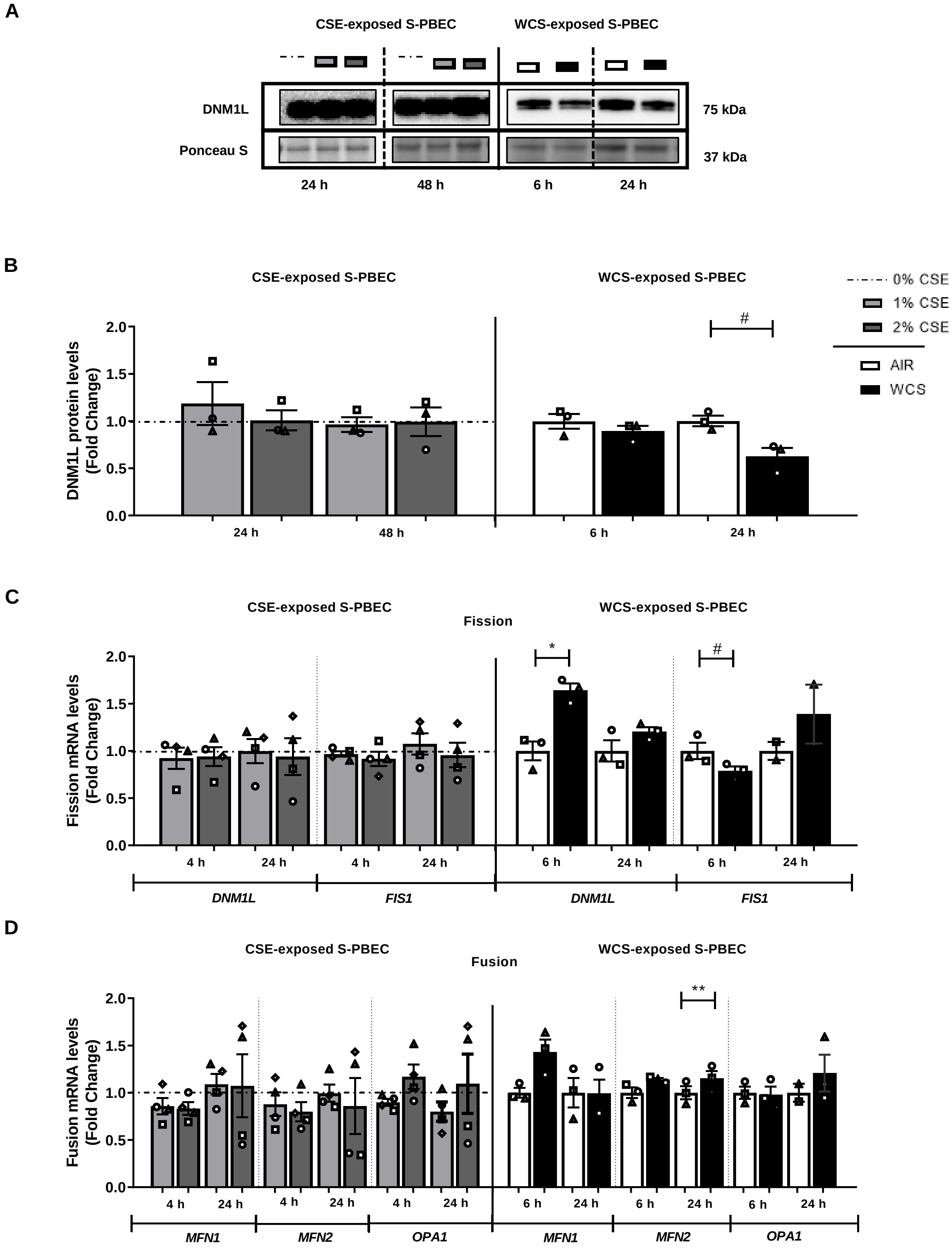

Disease Models & Mechanisms • Supplementary information

Fig. S9. Aberrant protein and transcript abundance of fission- and fusion-associated markers in S-PBEC. Undifferentiated S-PBEC were treated with CSE from one 3R4F cigarette (University of Kentucky) diluted in HBSS (0-1-2%) in Lonza starvation medium for 4 h, 24 h or 48 h (n=3-4 donors/group) or undifferentiated S-PBEC cultured on transwells were exposed, after removal of apical medium, to fresh air or WCS from one 3R4F cigarette (University of Kentucky, 2 mg) followed by harvesting of whole-cell lysates after 6 h or 24 h recovery (n=2-3 donors/group). Protein **(A, B)** and mRNA levels **(C)** of fission-associated markers (DNM1L, FIS1) and gene expression of fusion-associated markers (MFN1, MFN2, OPA1) **(D)** were measured using western blot and real-time qPCR. Representative western blots, including representative parts of the Ponceau S staining, are shown. Data are presented as mean fold change compared to control (0% CSE or air) ± s.e.m.. Independent donors are represented by open circles, triangles, squares or diamonds. In case of the CSE-exposed S-PBEC experiments, the symbols reflect the mean of technical triplicates. Statistical differences between the various CSE exposure groups (CSE 1% or 2% versus 0% CSE) were tested using an one-way ANOVA (matched/repeated measures) followed by Sidak's post-hoc test for multiple comparisons, and in case of missing values the mixed-effects models was performed. WCS versus air was tested using a two-tailed paired parametric *t*-test. Statistical significance is indicated as $^{\#}p<0.1$, $^{*}p<0.05$ and $^{**}p<0.01$ compared to control (0% CSE or air).

Disease Models & Mechanisms • Supplementary information

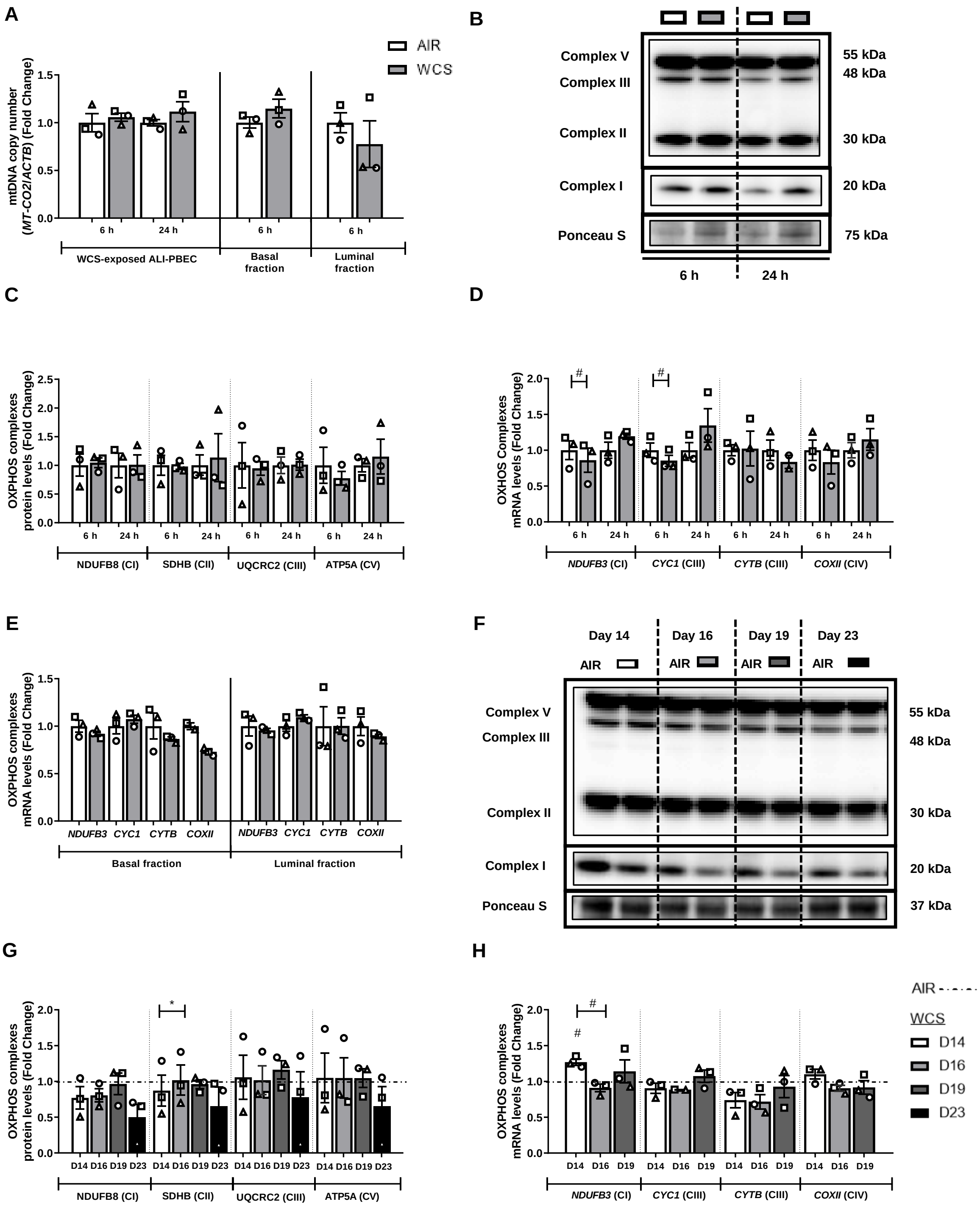

Disease Models & Mechanisms • Supplementary information

Fig. S10. Unaltered abundance of subunits of the electron transport chain in WCS-exposed ALI-PBEC. After 2-weeks of differentiation, ALI-PBEC were exposed to fresh air or WCS from one 3R4F cigarette (University of Kentucky, 2 mg) and whole-cell lysates were harvested after 6h and 24 h, and the basal and luminal fractions were harvested only at 6 h post-exposure (n=2-3 donors/group). Mitochondrial DNA copy number **(A)**, protein **(B, C)** as well as transcript levels **(D, E)** of nuclear and mitochondrial-encoded subunits of the electron transport chain (Complex I (CI), Complex II (CII), Complex III (CIII), Complex IV (CIV), Complex V (CV)) were analyzed in whole-cell lysates or basal and luminal fractions post-exposure. Data are presented as mean fold change compared to control (air) ± s.e.m.. Independent donors are represented by open circles, triangles or squares. Statistical differences between WCS versus air were tested using a two-tailed paired parametric *t*-test, $^{\#}p<0.1$. ALI-PBEC were 1x daily exposed to fresh air or WCS from one 3R4F cigarette (University of Kentucky, 2 mg) during differentiation for 14 days followed by a cessation period up to 10 days. Cells were harvested on Day 14 (24 h after the last exposure), 16, 19 and 23 (n=2-3 donors/group). Protein **(F, G)** and mRNA expression **(H)** of subunits of the electron transport chain are analyzed. Representative western blots, including representative parts of the Ponceau S staining, are shown. Data are presented as mean fold change compared to control (air or WCS Day 14) ± s.e.m.. Independent donors are represented by open circles, triangles or squares. Statistical differences between WCS versus air after smoking cessation in ALI-PBEC on each day was tested using a two-tailed paired parametric *t*-test (e.g, WCS Day 14 versus air). Comparison of various groups to test the difference of WCS Day 16, 19, 23 versus Day 14 in WCS chronic smoking cessation experiments was conducted using an one-way ANOVA followed by Sidak’s post-hoc test for multiple comparisons, and in case of missing values the mixed-effects models was performed. Statistical significance is indicated as $^{\#}p<0.1$ and $^{*}p<0.05$ compared to control (air or WCS Day 14).

Disease Models & Mechanisms • Supplementary information

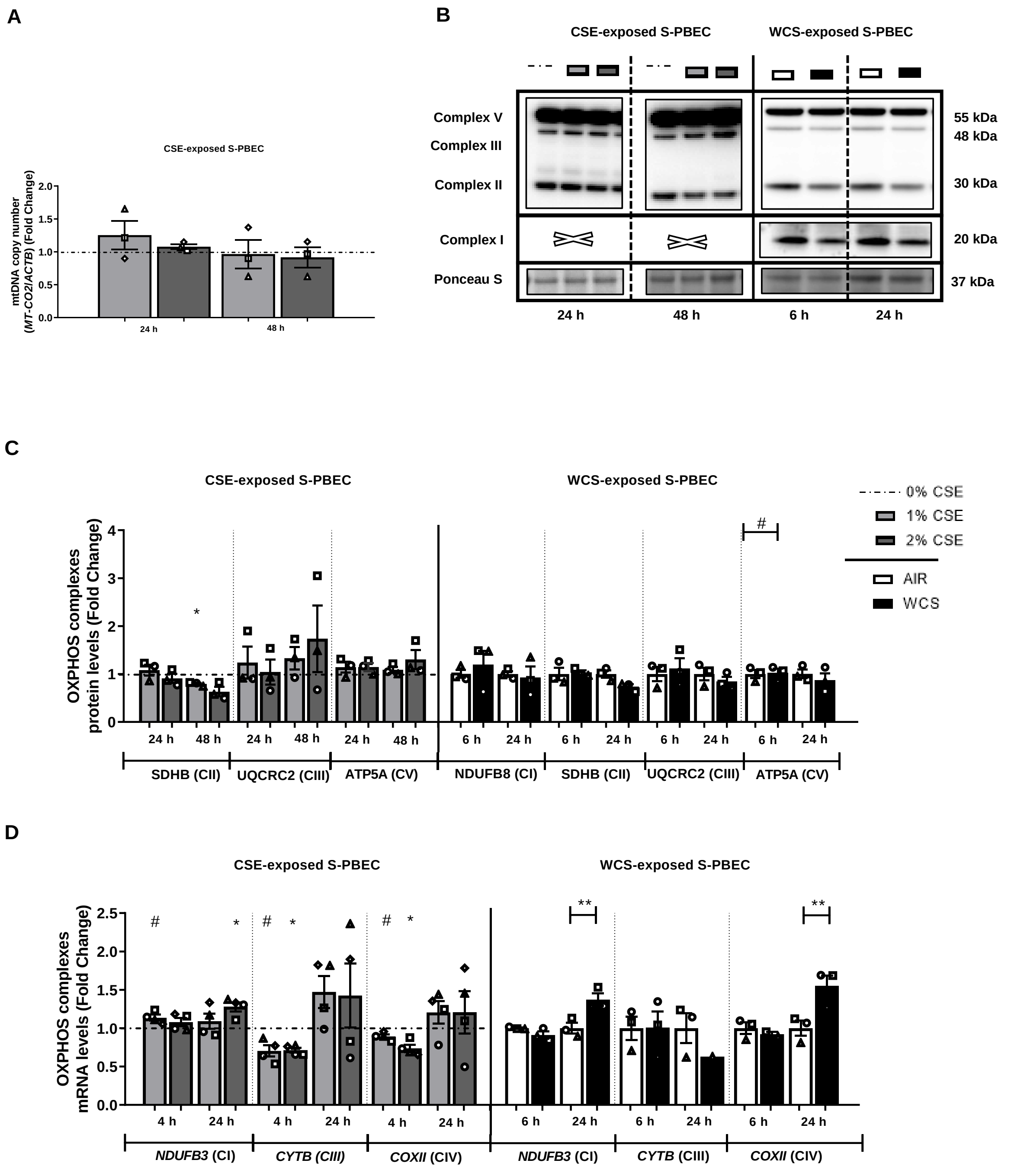

Disease Models & Mechanisms • Supplementary information

Fig. S11. Modulation in the abundance of subunits of the electron transport chain in CS-exposed S-PBEC. Undifferentiated S-PBEC were treated with CSE from one 3R4F cigarette (University of Kentucky) diluted in HBSS (0-1-2%) in Lonza starvation medium for 4 h, 24 h or 48 h (n=3-4 donors/group) or undifferentiated S-PBEC cultured on transwells were exposed, after removal of apical medium, to fresh air or WCS from one 3R4F cigarette (University of Kentucky, 2 mg) followed by harvesting of whole-cell lysates after 6 h or 24 h recovery (n=1-3 donors/group). Mitochondrial DNA copy number **(A)**, protein **(B, C)** as well as transcript levels **(D)** of nuclear and mitochondrial-encoded subunits of the electron transport chain (Complex I (CI), Complex II (CII), Complex III (CIII), Complex IV (CIV), Complex V (CV)) were analyzed in whole-cell lysates. Representative western blots, including representative parts of the Ponceau S staining, are shown. Data are presented as mean fold change compared to control (0% CSE or air) ± s.e.m.. Independent donors are represented by open circles, triangles, squares or diamonds. In case of the CSE-exposed S-PBEC experiments, the symbols reflect the mean of technical triplicates. Statistical differences between the various CSE exposure groups (CSE 1% or 2% versus 0% CSE) were tested using an one-way ANOVA (matched/repeated measures) followed by Sidak's post-hoc test for multiple comparisons, and in case of missing values the mixed-effects models was performed. WCS versus air was tested using a two-tailed paired parametric *t*-test. Statistical significance is indicated as $^{\#}p<0.1$, $^{*}p<0.05$ and $^{**}p<0.01$ compared to control (0% CSE or air).

Disease Models & Mechanisms • Supplementary information

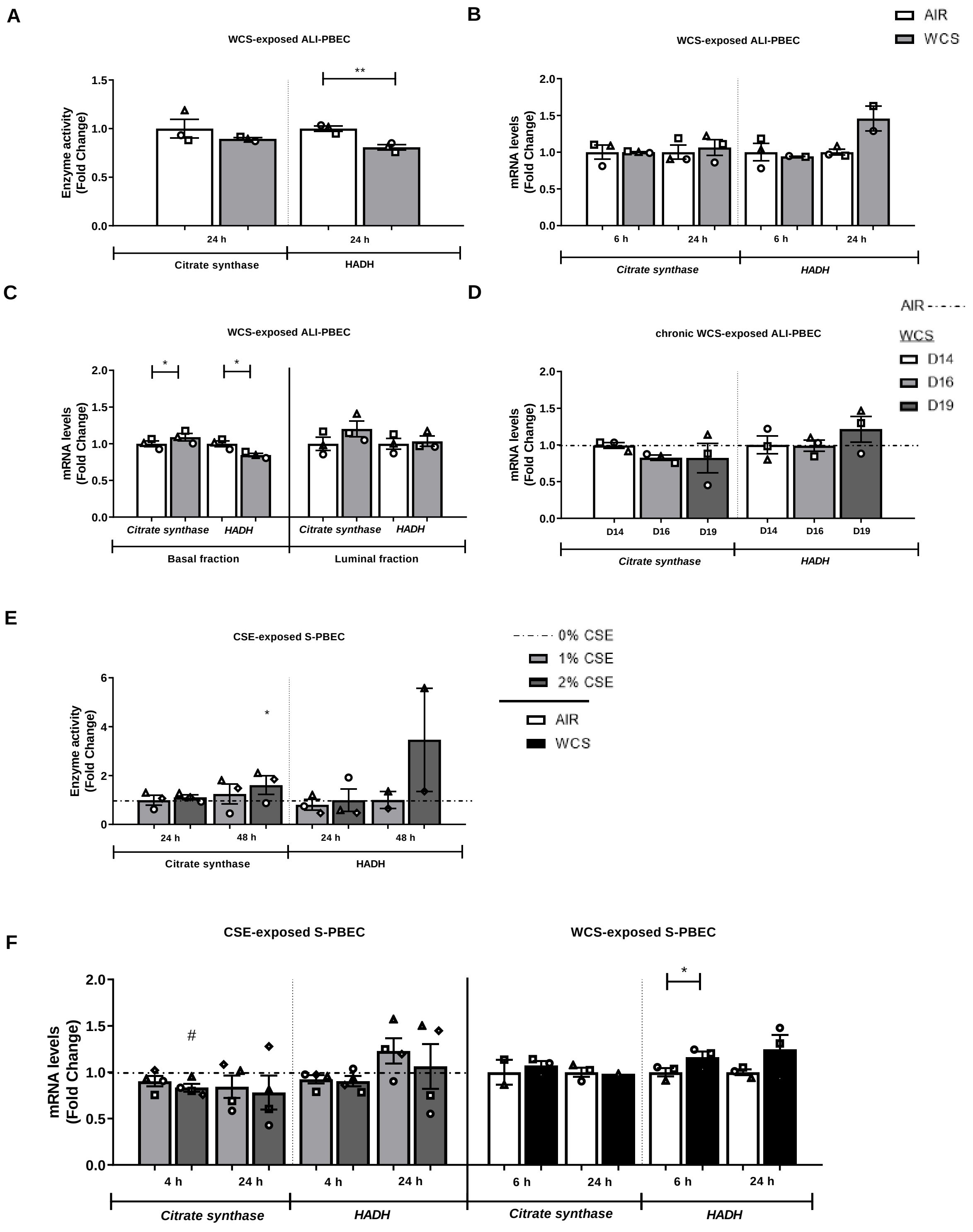
A
WCS-exposed ALI-PBEC
Enzyme activity (Fold Change)
24 h
24 h
Citrate synthase
HADH
B
AIR
WCS
WCS-exposed ALI-PBEC
mRNA levels (Fold Change)
6 h
24 h
6 h
24 h
Citrate synthase
HADH
C
WCS-exposed ALI-PBEC
mRNA levels (Fold Change)
Citrate synthase
HADH
Citrate synthase
HADH
Basal fraction
Luminal fraction
D
AIR
WCS
D14
D16
D19
chronic WCS-exposed ALI-PBEC
mRNA levels (Fold Change)
Citrate synthase
HADH
E
CSE-exposed S-PBEC
0% CSE
1% CSE
2% CSE
AIR
WCS
Enzyme activity (Fold Change)
24 h
48 h
24 h
48 h
Citrate synthase
HADH
F
CSE-exposed S-PBEC
WCS-exposed S-PBEC
mRNA levels (Fold Change)
4 h
24 h
4 h
24 h
6 h
24 h
6 h
24 h
Citrate synthase
HADH
Citrate synthase
HADH

Fig. S12. Disruption of activity and abundance of key components involved in the fatty acid β-oxidation in CS-exposed ALI- and S-PBEC. After 2-weeks of differentiation, ALI-PBEC were exposed to fresh air or WCS from one 3R4F cigarette (University of Kentucky, 2 mg) and whole-cell lysates were harvested after 6h and 24 h, and the basal and luminal fractions were harvested only at 6 h post-exposure (n=2-3 donors/group). Enzyme activities of citrate synthase and HADH **(A)** and related transcript abundance in whole-cell lysates **(B)** or basal and luminal fractions **(C)** were assessed in ALI-PBEC. Next, ALI-PBEC were 1x daily exposed to fresh air or WCS from one 3R4F cigarette (University of Kentucky, 2 mg) during differentiation for 14 days followed by a cessation period up to 10 days. Cells were harvested on Day 14 (24 h after the last exposure), 16 and 19 (n=3 donors/group). Transcript abundance **(D)** of *citrate synthase* and *HADH* were measured. Undifferentiated S-PBEC were treated with CSE from one 3R4F cigarette (University of Kentucky) diluted in HBSS (0-1-2%) in Lonza starvation medium for 4 h, 24 h or 48 h (n=4 donors/group) or undifferentiated S-PBEC cultured on transwells were exposed, after removal of apical medium, to fresh air or WCS from one 3R4F cigarette (University of Kentucky, 2 mg) followed by harvesting of whole-cell lysates after 6 h or 24 h recovery (n=1-3 donors/group). Cell lysates were used to measure enzyme activities of citrate synthase and HADH **(E)** and related transcript abundance **(F)**. Data are presented as mean fold change compared to control (air, 0% CSE or WCS Day 14) ± s.e.m.. Independent donors are represented by open circles, triangles, squares or diamonds. In case of the CSE-exposed S-PBEC experiments, the symbols reflect the mean of technical triplicates. Statistical differences between WCS versus air or WCS versus air after smoking cessation in ALI-PBEC on each day (e.g., WCS Day 14 versus air) were tested using a two-tailed paired parametric *t*-test. If comparison of various groups was required in case of the CSE exposure (CSE 1% or 2% versus 0% CSE) or in WCS chronic smoking cessation experiments (WCS Day 16, 19 versus WCS Day 14), an one-way ANOVA (matched/repeated measures) followed by Sidak's post-hoc test for multiple comparisons was conducted, and in case of missing values the mixed-effects models was performed. Statistical significance is indicated as $^{\#}p<0.1$, $^{*}p<0.05$ and $^{**}p<0.01$ compared to control (air, 0% CSE or WCS Day 14).

Disease Models & Mechanisms • Supplementary information

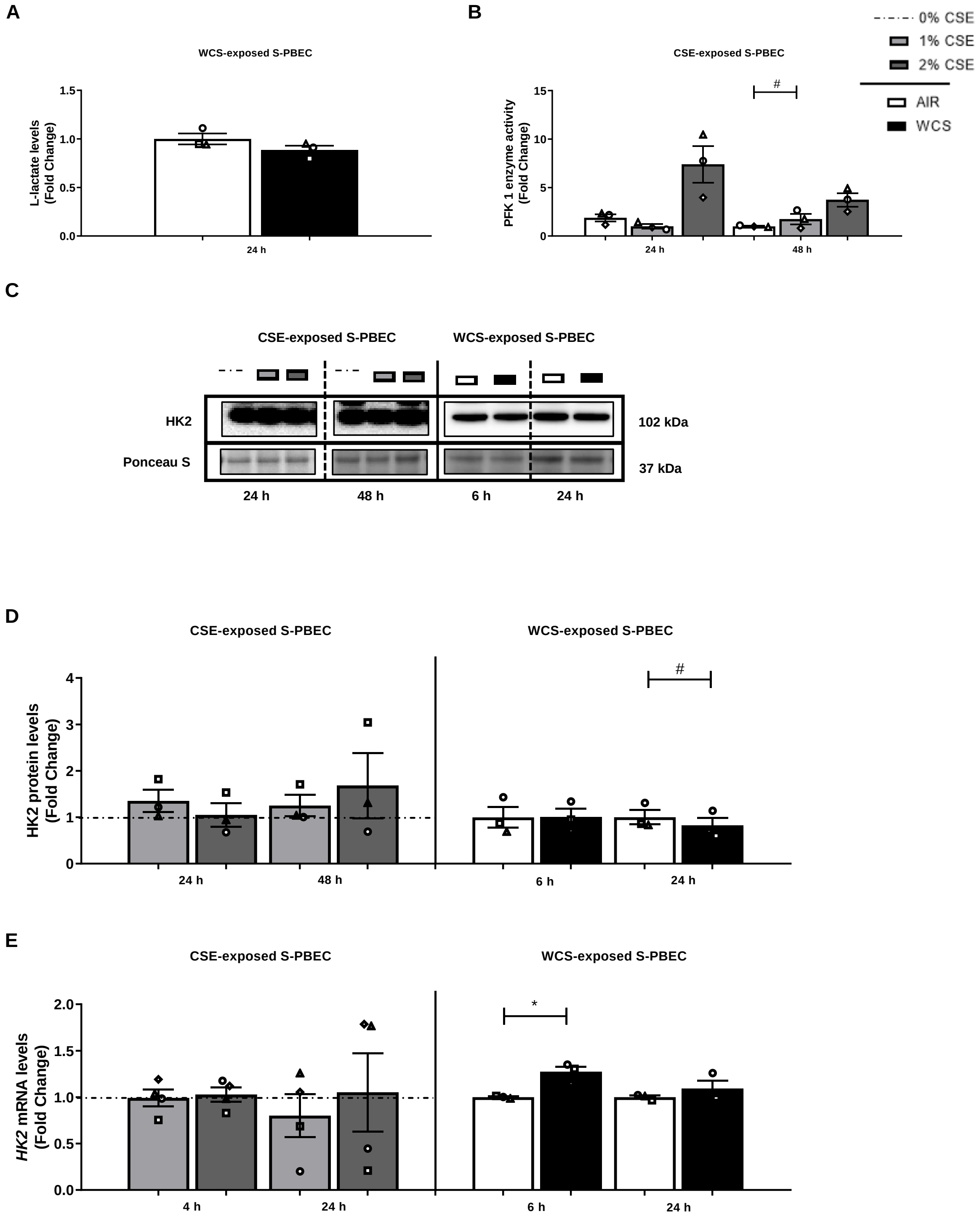

Disease Models & Mechanisms • Supplementary information

Fig. S13. Glycolytic shift after CS exposure in S-PBEC. Undifferentiated S-PBEC were treated with CSE from one 3R4F cigarette (University of Kentucky) diluted in HBSS (0-1-2%) in Lonza starvation medium for 4 h, 24 h or 48 h (n=3-4 donors/group) or undifferentiated S-PBEC cultured on transwells were exposed, after removal of apical medium, to fresh air or WCS from one 3R4F cigarette (University of Kentucky, 2 mg) followed by harvesting of whole-cell lysates after 6 h or 24 h recovery (n=3 donors/group). L-lactate levels **(A)**, PFK 1 enzyme activity **(B)** as well as protein **(C, D)** and mRNA levels **(E)** of HK2 were analyzed in S-PBEC. Representative western blots, including representative parts of the Ponceau S staining, are shown. Data are presented as mean fold change compared to control (0% CSE or air) ± s.e.m.. Independent donors are represented by open circles, triangles, squares or diamonds. In case of the CSE-exposed S-PBEC experiments, the symbols reflect the mean of technical triplicates. WCS versus air was tested using a two-tailed paired parametric *t*-test. Statistical differences between the various CSE exposure groups (CSE 1% or 2% versus 0% CSE) were tested using an one-way ANOVA (matched/repeated measures) followed by Sidak's post-hoc test for multiple comparisons, and in case of missing values the mixed-effects models was performed. Statistical significance is indicated as $^{\#}p<0.1$ and $^{*}p<0.05$ compared to control (0% CSE or air).

Table S1. Human primers sequences used for real-time quantitative PCR analysis.

Gene	Sense primer (5'-3')	Antisense primer (3'-5')
**Reference genes**		
ATP5B	TCACCCAGGCTGGTTCAGA	AGTGGCCAGGGTAGGCTGAT
B2M	CTGTGCTCGCGCTACTCTCTCTT	TGAGTAAACCTGAATCTTTGGAGTACGC
ACTB	AAGCCACCCCACTTCTCTCTAA	AATGCTATCACCTCCCCTGTGT
PPIA	CATCTGCACTGCCAAGACTGA	TTCATGCCTTCTTTCACTTTGC
RPL13A_1	CCTGGAGGAGAAGAGGAAAGAGA	TTGAGGACCTCTGTGTATTTGTCAA
RPL13A_2	AAGGTGGTGGTCGTACGCTGTG	CGGGAAGGGTTGGTGTTCATCC
**Target genes**		
BNIP3	AGCGCCCGGGATGCA	CCCGTTCCCATTATTGCTGAA
BNIP3L	CTGCGAGGAAAATGAGCAGTCTCT	GCCCCCCATTTTTCCCATTG
Citrate synthase	GATGTGTCAGATGAGAAGTTACGAGACT	TGGCCATAGCCTGGAACAA
COXII	ACCTGCGACTCCTTGACGTT	GGGGGCTTCAATCGGGAGTA
CYC1	GAGCACGACCATCGAAAACG	CGATATGCCAGCTTCCGACT
DNM1L	CGACTCATTAAATCATATTTTCTCATTGTCAG	TGCATTACTGCCTTTGGCACACT
ESRRA	TGCTGCTCACGCTACCGCTC	TCGAGCATCTCCAAGAACAGC
FIS1	CCTGGTGCGGAGCAAGTACAA	TCCTTGCTCCCTTTGGGCAG
FUNDC1	GAAACGAGCGAACAAAGCAG	GCAAAAAGCCTCCCACAAAT
GABARPL1	ATCGGAAAAAGGAAGGAGAAAAGATC	CAGGCACCCTGGCTTTTGG
HADHA	TGGCTTCCCGCCTTGTC	TGGAGCCGGTCCACTATCTTC
HK2	GTAAATACAGTGGATCTCAATCTTCGGG	CAAGGATTTGAGATGATTCGCTATTCA
KRT8	TCCTCAGGCAGCTATATGAAGAG	GGTTGGCAATATCCTCGTACTGT
MAP1LC3A	CCTGGACAAGACCAAGTTTTTG	GTCTTTCTCCTGCTCGTAGATG
MAP1LC3B	ACCATGCCGTCGGAGAAGAC	TCTCGAATAAGTCGGACATCTTCTACTCT
MFN1	CTGAGGATGATTGTTAGCTCCACG	CAGGCGAGCAAAAGTGGTAGC

Disease Models & Mechanisms • Supplementary information

MFN2	TGGACCACCAAGGCCAAGGA	TCTCGCTGGCATGCTCCAC
Mt-CytB	ACCCCCTAGGAATCACCTCC	GCCTAGGAGGTCTGGTGAGA
NDUFB3	TCAGATTGCTGTCAGACATGG	TGGTGTCCCTTCTATCTTCCA
NRF1	AGGAACACGGAGTGACCCAA	TATGCTCGGTGTAAGTAGCCA
OPA1	TACCAAAGGCATTTTGTAGATTCTGAGTT	GCATGCGCTGTATACGCCAA
PIGR	CTCTCTGGAGGACCACCGT	CAGCCGTGACATTCCCTG
PINK1	GAAAGCCGCAGCTACCAAGA	AGCACATTTGCGGCTACTCG
PPARGC1A	AAGCCACTACAGACACCGC	TCGTAGCTGTCATACCTGGG
PPARGC1B	GGCGCTTTGAAGTGTTTGGTGA	TGATGAAGCCGTACTTCTCGCCT
PPRC1	GCCCTTTGATCTCTGCTTTGGG	AAGTCTTCCCGGTTGGAGTCAAG
PRKN	GGTTTGCCTTCTGCCGGGAATG	CTTTCATCGACTCTGTAGGCCTG
SDHB	TGGGGCCTGCAGTTCTTATG	ATGGTGTGGCAGCGGTATAG
**Gene**	**Sense primer (5’-3’)**	**Antisense primer (3’-5’)**
**Target genes**		
SOD1	GGTCCTCACTTTAATCCTCTAT	CATCTTTGTCAGCAGTCACATT
SOD2	TGGACAAACCTCAGCCCTAACG	TGATGGCTTCCAGCAACTCCC
SQSTM1	GGTGCACCCCAATGTGATCT	CGCAGACGCTACACAAGTCG
TFAM	GAAAGATTCCAAGAAGCTAAGGGTGATT	TCCAGTTTTCCTTTACAGTCTTCAGCTTTT
TP63	CCACCTGGACGTATTCCACTG	TCGAATCAAATGACTAGGAGGGG

Abbreviations:

ATP5B: ATP synthase F1 subunit beta, *B2M*: beta-2 microglobulin, *ACTB*: actin B, *PPIA*: peptidylprolyl isomerase A, *RPL13A*: Ribosomal Protein L13A, *BNIP3*: BCL2 interacting protein 3, *BNIP3L*: BCL2 interacting protein 3-like, Citrate synthase, *COXII*: Cytochrome c oxidase subunit II, *CYC1*: cytochrome c-1, *DNM1L*: dynamin 1-like, *ESRRA*: estrogen related receptor, alpha, *FIS1*: fission, mitochondrial 1, *FUNDC1*: FUN14 domain containing 1, *GABARAPL1*: GABA type A receptor associated protein like 1, *HADHA*: hydroxyacyl-CoA dehydrogenase trifunctional

Disease Models & Mechanisms • Supplementary information

multienzyme complex subunit alpha, *HK2*: hexokinase 2, *KRT8*: Keratin 8, *MAP1LC3A*: microtubule-associated protein 1 light chain 3 alpha, *MAP1LC3B*: microtubule-associated protein 1 light chain 3 beta, *MFN1*: mitofusin 1, *MFN2*: mitofusin 2, *Mt-CytB*: Mitochondrial-encoded Cytochrome Beta, *NDUFB3*: NADH:ubiquinone oxidoreductase subunit B3, *NRF1*: nuclear respiratory factor 1, *OPA1*: OPA1, mitochondrial dynamin like GTPase, *PIGR:* Polymeric Immunoglobulin Receptor, *PINK1*: PTEN Induced Kinase 1, *PPARGC1A*: PPARG coactivator 1 alpha, *PPARGC1B*: PPARG coactivator 1 beta, *PPRC1*: PPARG related coactivator 1, *PRKN*: Parkin RBR E3 Ubiquitin Protein Ligase, *SDHB*: succinate dehydrogenase complex iron sulfur subunit B, *SOD1*: superoxide dismutase 1, *SOD2*: superoxide dismutase 2, *SQSTM1*: sequestosome 1, *TFAM*: transcription factor A, mitochondrial, *TP63*: tumor protein 63

Disease Models & Mechanisms • Supplementary information

Table S2. Antibodies used for western blotting

Target	RRID	Company	Product number	Dilution factor
**Primary Antibodies**				
BNIP3	AB_2259284	Cell Signaling Technology	Cat# 3769S	1:1000
BNIP3L	AB_2688036	Cell Signaling Technology	Cat# 12396	1:1000
DNM1L	AB_10950498	Cell Signaling Technology	Cat# 8570	1:1000
ESRRA	AB_1523580	Abcam	Cat# ab76228	1:1000
FUNDC1	AB_10609242	Santa Cruz Biotechnology	Cat# sc-133597	1:500
GABARAPL1	AB_2294415	Proteintech Group	Cat# 11010-1-AP	1:1000
HK2	AB_2232946	Cell Signaling Technology	Cat# 2867	1:1000
MAP1LC3B	AB_915950	Cell Signaling Technology	Cat# 2775	1:1000
NRF1	AB_2154534	Abcam	Cat# ab55744	1:1000
OXPHOS	AB_2629281	MitoScience LLC	Cat# MS604	1:1000
PINK1	AB_10127658	Novus Biologicals	Cat# BC100-494	1:2000
PPARGC1A	AB_10697773	Millipore	Cat# 516557	1:1000
PRKN	AB_2159920	Cell Signaling Technology	Cat# 4211	1:1000
SQSTM1	AB_10624872	Cell Signaling Technology	Cat# 5114	1:1000
TFAM	AB_10682431	Millipore	Cat# DR1071	1:1000
**Secondary Antibodies**				
Goat Anti-Mouse IgG Antibody	AB_2827937	Vector Laboratories	Cat#BA-9200	1:10000
Goat Anti-Rabbit IgG Antibody	AB_2313606	Vector Laboratories	Cat#BA-1000	1:10000

Disease Models & Mechanisms • Supplementary information

Abbreviations:

BNIP3: BCL2 Interacting Protein 3, BNIP3L: BCL2 Interacting Protein 3-Like, DNM1L: dynamin 1-like, ESRRA: Estrogen Related Receptor, Alpha, FUNDC1: FUN14 Domain Containing 1, GABARAPL1: GABA Type A Receptor Associated Protein Like 1, HK2: Hexokinase 2, MAP1LC3B (or LC3B): Microtubule-Associated Protein 1 Light Chain 3 Beta, NRF1: Nuclear Respiratory Factor 1, OXPHOS: oxidative phosphorylation antibody cocktail (containing NDUFB8: NADH:Ubiquinone Oxidoreductase Subunit B8, SDHB: Succinate Dehydrogenase Complex Iron Sulfur Subunit B, UQCRC2: Ubiquinol-Cytochrome C Reductase Core Protein 2, MT-COI: Mitochondrially Encoded Cytochrome C Oxidase I, ATP5F1A: ATP Synthase F1 Subunit Alpha), PINK1: PTEN Induced Kinase 1, PPARGC1A: PPARG Coactivator 1 Alpha, PRKN: Parkin RBR E3 Ubiquitin Protein Ligase, SQSTM1: Sequestosome 1, TFAM: Transcription factor A, Mitochondrial

Disease Models & Mechanisms • Supplementary information
